# Performance-limiting formation dynamics in mixed-halide perovskites

**DOI:** 10.1126/sciadv.abj1799

**Published:** 2021-11-10

**Authors:** Tianyi Huang, Shaun Tan, Selbi Nuryyeva, Ilhan Yavuz, Finn Babbe, Yepin Zhao, Maged Abdelsamie, Marc H. Weber, Rui Wang, Kendall N. Houk, Carolin M. Sutter-Fella, Yang Yang

**Affiliations:** 1 Department of Materials Science and Engineering and California NanoSystems Institute, University of California Los Angeles, Los Angeles, CA 90095, USA.; 2 Chemical Sciences Division, Lawrence Berkeley National Laboratory, Berkeley, CA 94720, USA.; 3 Department of Chemistry, University of California, Los Angeles, CA 90095, USA.; 4 Department of Physics, Marmara University, 34722 Ziverbey, Istanbul, Turkey.; 5 Materials Sciences Division, Lawrence Berkeley National Laboratory, Berkeley, CA 94720, USA.; 6 Center for Materials Research, Washington State University, Pullman, WA 99164, USA.; 7 Molecular Foundry, Lawrence Berkeley National Laboratory, Berkeley, CA 94720, USA.

## Abstract

Wide-bandgap (WBG) mixed-halide perovskites as the front cell absorber are accomplishing perovskite-based tandem solar cells with over 29% power conversion efficiency. However, their large voltage deficits limit their ultimate performance. Only a handful of studies probe the fundamental mechanisms underlying the voltage deficits, which remain an unsolved challenge in the field. In this study, we investigate the formation dynamics and defect physics of WBG mixed-halide perovskites in contrast with their corresponding triiodide-based perovskites. Our results show that the inclusion of bromide introduced a halide homogenization process that occurs during the perovskite growth stage from an initial bromide-rich phase toward the final target stoichiometry. We further elucidated a physical model that correlates the role of bromide with the formation dynamics, defect physics, and eventual optoelectronic properties of the film. This work provides a fundamental and unique perspective toward understanding the performance-limiting factors affecting WBG mixed-halide perovskites.

## Teaser

Bromide inclusion altered the formation dynamics and defect physics of wide-bandgap perovskite compositions.

## INTRODUCTION

Metal halide perovskites with their X-sites of iodine partially substituted with bromine have demonstrated great potential for commercialization of halide perovskite technology for use in tandem photovoltaics (PVs) integrated with conventional PV products such as Si and CuInGaSe_2_ (CIGS) (*1*). By controlling the I/Br ratio, the optical bandgaps of mixed-halide perovskites can be tuned to be between 1.64 and 1.70 eV, usually referred to as wide-bandgap (WBG) perovskites in the field, ideal for front-cell applications in two-junction tandem PVs (*2*). With such WBG mixed-halide perovskites, perovskite-Si and perovskite-CIGS tandem cells have reached remarkable power conversion efficiencies (PCEs) of 29.5 and 24.2%, respectively (*3*).

Conversely, single-junction PVs based on most FAPbI_3_ triiodide perovskite compositions (*E*_g_ = 1.48 eV, *V*_OC, SQ_ = 1.21 V, and *V*_OC, reported_ = 1.18 V) have recently exceeded 25% PCE, with voltage deficits, defined as the difference between the optical bandgap and open-circuit voltage (*V*_OC_), as low as 0.30 V (*4*). For WBG mixed-halide perovskites, however, currently, even the best reported subcell with hole transporting materials with negligible energy offsets with the perovskite layer exhibited a voltage deficit up to 0.46 V (*E*_g_ = 1.68 eV, *V*_OC, SQ_ = 1.40 V, and *V*_OC, reported_ = 1.22 V) (*5*). Therefore, the voltage deficits in WBG perovskites continue to lag their corresponding single-junction counterparts. Active debate is ongoing regarding the mechanistic reasons underlying the large voltage deficits in WBG perovskites, which remains an unsolved challenge in the field. Arguably, the voltage deficits remain the largest bottleneck toward further improving the performance of perovskite-based tandem PVs.

Voltage deficits are directly correlated with nonradiative energy losses. Although halide perovskites are reported to have high defect tolerance (*6*, *7*), deep traps still do exist, especially toward the top surface region (*8*), that may induce trap-mediated recombination losses (*9*). Intrinsic defects form during the nucleation and growth stages of the perovskite crystals from solution, and their formation is highly dependent on several processing conditions and especially the precursor stoichiometry and composition. On this note, since the cation itself minimally affects the optical bandgap, cation engineering has been intensively studied and explored. For example, Qin *et al*. (*10*) reported that formulations with Cs^+^ markedly affected the perovskite formation dynamics and processing window, which was subsequently found by Correa-Baena *et al.* (*11*) to be also essential to achieve homogeneous halide distribution and is critical for highly efficient mixed-halide perovskite PVs. These pioneering works have provided many insights into the cations’ role in preparing mixed-halide perovskites and particularly their effects on achieving halide homogeneity and preventing halide segregation during operation (*5*, *12*–*14*). However, given that the halide ratio in WBG compositions is mostly preserved to achieve the necessary optical bandgap, the role of the anion on the perovskite formation dynamics has been largely unexplored. The I/Br ratio may potentially alter the nucleation and growth behaviors of WBG perovskites, which may further be related to the defect physics and optoelectronic properties of the films.

In this work, we investigated the role of Br mixing on the formation dynamics of WBG perovskites. Combining systematic experimental studies with first-principle calculations, our observations suggest that the representative FAMACsPb(I_0.8_Br_0.2_)_3_ perovskite (FA=Formamidinium, MA=Methylammonium, and Cs=Cesium) undergoes a complex crystallization pathway by first nucleating a Br-rich phase from solution during supersaturation before experiencing a retarded growth stage during perovskite growth. The slow growth stage is associated with a halide homogenization process that brings the lattice composition to the eventual target stoichiometry. This homogenization process inadvertently altered the formation dynamics of mixed-halide perovskites and promoted defect formation, which consequently led to increased nonradiative recombination losses in the final perovskite film. In other words, Br affected the perovskite formation dynamics, which may have contributed to the large voltage deficits in high Br%-containing compositions.

## RESULTS

**Fig. 1.**
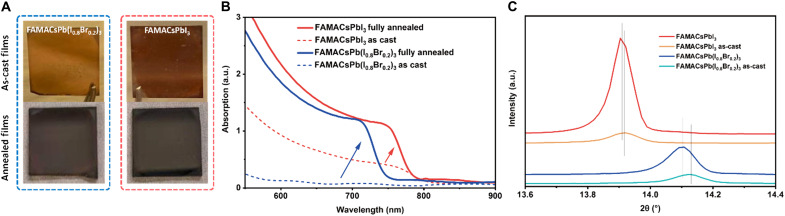
Abnormal formation dynamics in WBG mixed-halide perovskites. (**A**) Photographs of the as-cast (top) and fully annealed (bottom) perovskite films with the stoichiometries of CsFAMAPb(I_0.8_Br_0.2_)_3_ (left) and CsFAMAPbI_3_ (right). (**B**) Absorption spectra of FAMACsPbX_3_ films before (as-cast)/after fully annealed. (**C**) XRD spectra of (001) peak of FAMACsPbX_3_ films. a.u., arbitrary units.

We specifically focused on the triple cation mixed-halide WBG perovskite FAMACsPb(I_0.8_Br_0.2_)_3_, as it is one of the most widely reported compositions with a good resistance against light-induced halide segregation and also high PV performance. The triiodide reference with the same triple cations, FAMACsPbI_3_, will serve as the reference to investigate the effects of Br inclusion on the perovskite formation dynamics. As shown in both the digital photographs (Fig. 1A) and absorption spectra (Fig. 1B) of the two perovskites, the as-cast FAMACsPbI_3_ film already exhibited strong band edge absorption, indicating that the α-phase perovskite has partially formed. In contrast, the as-cast FAMACsPb(I_0.8_Br_0.2_)_3_ film was more transparent and showed negligible absorption associated with its perovskite phase near its target bandgap of approximately 1.67 eV (745 nm). After annealing, the band edge absorptions and absorption coefficients for both compositions were similar (but displaced in wavelength), hinting that the formation dynamics during crystallization have been altered by the inclusion of Br. We further investigated the films using x-ray diffraction (XRD). Both as-cast films exhibited the α-perovskite (001) peak. Upon annealing, the (001) peak position for FAMACsPb(I_0.8_Br_0.2_)_3_ underwent a notably larger shift toward lower 2θ (Fig. 1C and fig. S1), whereas the peak position for FAMACsPbI_3_ changed negligibly in comparison. Decreasing Br content in the perovskite lattice would increase the lattice constant (smaller 2θ); therefore, the XRD results perhaps suggest that a Br-rich phase nucleated initially, and the lattice Br ratio is decreasing during the growth stage due to gradual iodide incorporation into the perovskite nuclei during crystallization [the FACs (MA-free) system was also explored in detail; see figs. S2 to S3]. Furthermore, although the cation composition is fixed as a constant for the above-discussed perovskite films, we further analyzed the XRD patterns of MAPb(I*_x_*Br_1−*x*_)_3_, FAPb(I*_x_*Br_1−*x*_)_3_ (with or without MACl additive), FAMACsPb(I*_x_*Br_1−*x*_)_3_, and FACsPb(I*_x_*Br_1−*x*_)_3_ films to rule out any possible contribution by the cations and the MACl additive (figs. S5 to S9; detailed discussion in Supplementary Text), and the observations from these “single cation” and the “MACl-free” films were consistent with the triple-cation system with MACl that delivered the champion performance. Therefore, we hypothesized that the initial nucleation was most likely dominated by a Br-rich phase, which is followed by slower iodide incorporation during the growth stage. We also observed that PbI_2_ formed more readily in FAMACsPb(I_0.8_Br_0.2_)_3_, and the film crystallinity was poorer than FAMACsPbI_3_ (fig. S1; detailed discussion in Supplementary Text).

**Fig. 2.**
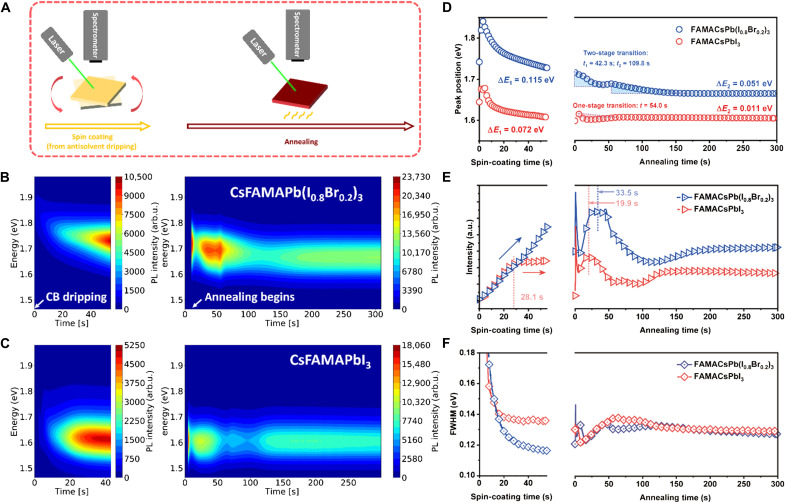
In situ PL measurements monitoring the formation kinetics of perovskite films. (**A**) Illustration of the in situ PL measurement during spin-coating and annealing stage of perovskite formation. The contour plot of the captured PL spectra during the growth of (**B**) CsFAMAPb(I_0.8_Br_0.2_)_3_ and (**C**) CsFAMAPbI_3_ films during spin coating (left) and annealing (right). The extracted values of emission peak position (**D**), PL intensity (**E**), and FWHM (**F**) from the in situ PL measurements.

To investigate such a hypothesis, we used in situ photoluminescence (PL) to monitor the perovskite crystallization process in real time. In contrast to diffraction methods, in situ PL provides direct proof of the bandgap evolution during growth, which is dominated mainly by the halide ratio in halide perovskites and thus is valuable to probe the formation dynamics related to the halide elements in real time. A 532-nm laser diode, coupled with a visible range spectrometer, was mounted in an N_2_ glove box to track the film photoemission during nucleation and growth. As shown in Fig. 2A, the experiment was designed to collect the PL signal during the approximately 1 min of spin-coating step, followed by annealing at 65°C for 5 min. Figure 2 (B and C) depicts the PL spectra evolution during the formation of the mixed-halide FAMACsPb(I_0.8_Br_0.2_)_3_ and triiodide FAMACsPbI_3_; data displayed on the left initiates from antisolvent dripping during spin coating. Figure S10 exemplifies the Gaussian fitting of the in situ PL spectra to extract the PL peak position, intensity, and full width at half maximum (FWHM), as plotted in Fig. 2 (D to F). It was observed that, for both formulations, their respective PL signals initiated at much higher energy levels [1.88 eV for FAMACsPb(I_0.8_Br_0.2_)_3_ and 1.67 eV for FAMACsPbI_3_] compared with the final bandgap of the bulk perovskite films (approximately 1.67 and 1.57 eV, respectively, from their PL maxima). For FAMACsPb(I_0.8_Br_0.2_)_3_, the energy shifted by 0.115 eV during the spin-coating stage (Δ*E*_1_) and 0.051 eV upon annealing (Δ*E*_2_). The corresponding values were 0.072 and 0.011 eV for FAMACsPbI_3_, much less than the mixed-halide composition. We speculated that the emission peak shifting during the perovskite formation, especially during the early growth period, could be attributed to a combination of two effects: (i) The quantum confinement of the nanograins, in which case a larger initial crystal size would show smaller energy shifting; and (ii) a compositional evolution during perovskite formation dominated by an initial higher bandgap species (*15*–*17*). Therefore, the larger Δ*E* in FAMACsPb(I_0.8_Br_0.2_)_3_ is most likely due to the formation of an initial Br-rich nuclei, complementing the XRD analysis discussed.

On the other hand, the FAMACsPbI_3_ film took only ~54 s to reach its stable emission peak position during annealing, while for FAMACsPb(I_0.8_Br_0.2_)_3_, the emission peak shifting lasted for over 150s. Therefore, the growth stage has been retarded in FAMACsPb(I_0.8_Br_0.2_)_3_, likely associated with an increased activation energy barrier for perovskite growth (*18*). The emission peak transition during annealing for FAMACsPb(I_0.8_Br_0.2_)_3_ underwent two separate stages (denoted as *t*_1_ beginning at 42.3 s and *t*_2_ beginning at 109.8 s) before its stabilization. We further compared the growth evolution with different amounts of Br incorporation of 20, 10, 5, and 0% (fig. S11), observing that the growth stage successively retarded further with increasing Br%. The PL intensity (Fig. 2E) observed during the growth process was a combination of several effects, including the formation of increasing amounts of highly illuminating perovskite phase crystals, countered by self-absorption as the perovskite amount increases, especially close to the band edge. As the size and specific area of crystalline perovskite change during growth, trap-assisted nonradiative recombination could also play a dominant role. Overall, we found that the PL intensity increases during the spin-coating stage, while it continued to increase to a maximum in tens of seconds, and then decreases before the signal stabilizes during the annealing stage. We speculate that the formation of illuminating perovskite crystals dominated the PL intensity during spin coating, while during annealing, the time required to reach the intensity maximum serves as an indicator of the crystal growth rate (i.e., the earlier it reaches the intensity maximum, the faster is the growth rate). For FAMACsPbI_3_, we observed that the signal quickly “saturated” after 28.1 s upon casting the antisolvent, while the intensity of FAMACsPb(I_0.8_Br_0.2_)_3_ continued to increase during spin coating (Fig. 2E, left slab). During the annealing stage, these two compositions reached their maximum intensities at 33.5 and 19.9 s, respectively. Analyzing the FWHM (Fig. 2F), the signal from FAMACsPb(I_0.8_Br_0.2_) achieved a much sharper peak during spin coating and stabilized slower than that of the FAMACsPbI_3_. For the fully annealed films shown in fig. S12, both compositions exhibited clear and monopeak PL, indicating that both films eventually reached their target stoichiometry with good phase homogeneity, and matched well with their absorption cutoffs. The bandgaps were determined to be approximately 1.56 and 1.67 eV for the as prepared FAMACsPbI_3_ and FAMACsPb(I_0.8_Br_0.2_)_3_ films, respectively. This indicates that a halide homogenization process must have occurred during the growth stage to achieve the target stoichiometry from the initial Br-rich phase. Such a homogenization process occurs by diffusion of iodide to incorporate into the initial Br-rich phase, which introduces an activation energy barrier and is consistent with a retardation of the perovskite growth (*19*). We further explored the FACs (MA-free) composition, which has also been reported to be highly resistant to halide segregation and delivers high PCE. The trends were consistent with the triple-cation system (figs. S13 and S14; for detailed discussion, see Supplementary Text), supporting our hypothesis and demonstrating the universal role of Br.

**Fig. 3.**
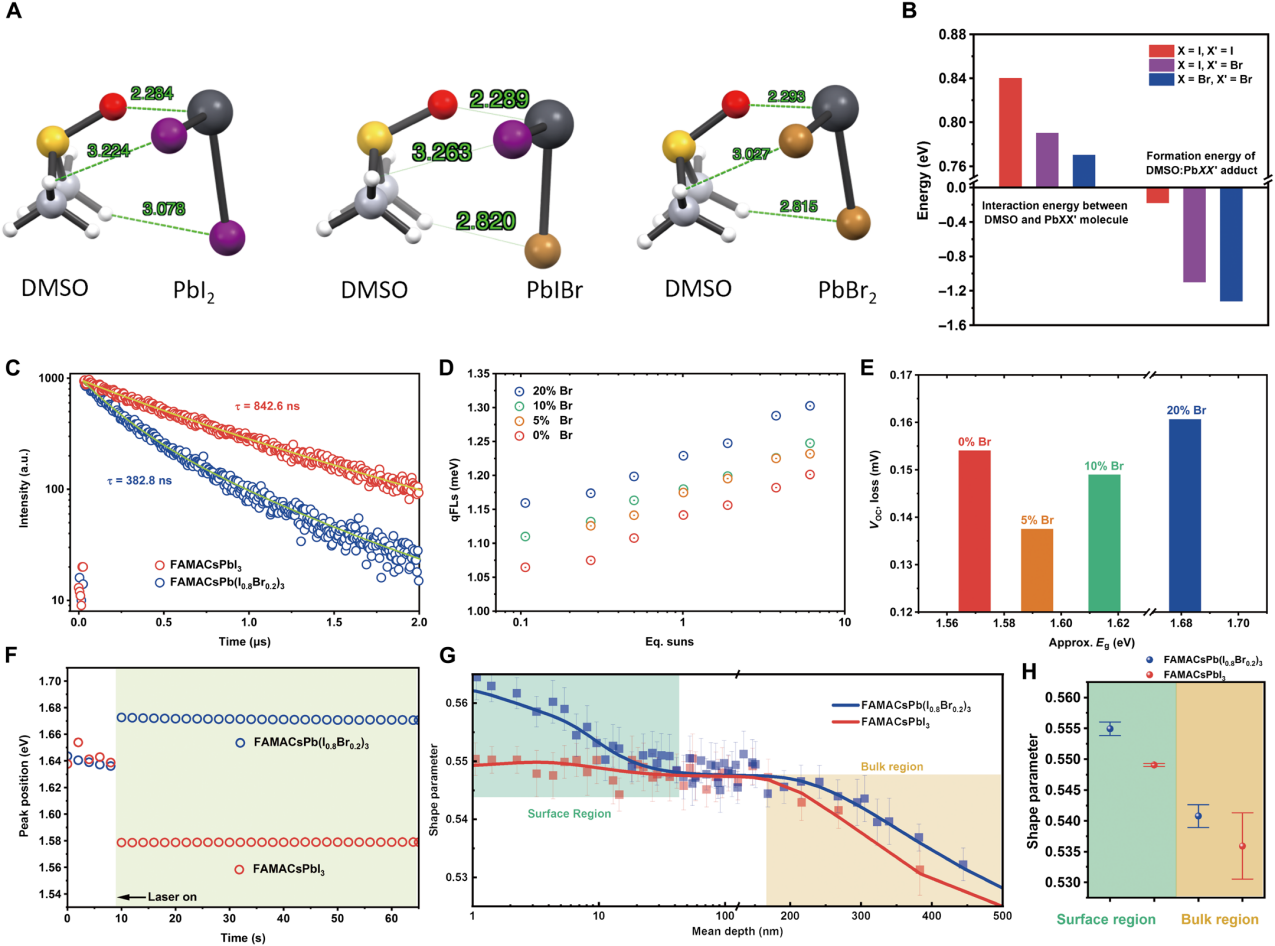
First-principle calculations and characterizations of the fully annealed perovskite films. (**A**) Molecular configuration and interaction distance of coordinating solvent DMSO and PbXX′ molecules. (**B**) Interaction energy between DMSO and PbXX′ molecule and formation energy of DMSO:PbXX′ adduct phase. (**C**) Time-resolved PL spectra of the as-prepared CsFAMAPb(I_0.8_Br_0.2_)_3_ and CsFAMAPbI_3_ films. (**D**) Quasi-Fermi level splitting (qFL) results quantified by a calibrated laser intensity and (**E**) the as-extracted *V*_OC,loss_ (*V*_OC,SQ_ − qFLs) for perovskite films with Br% of 20, 10, 5, and 0%. (**F**) The extracted values of emission peak position from 60-s PL tracking. (**G**) PAS depth profiling of CsFAMAPb(I_0.8_Br_0.2_)_3_ and CsFAMAPbI_3_ films. Solid lines are fitted plots. Green/orange shaded areas indicate the top surface/bulk region of the films. (**H**) Shape parameters from PAS extracted for the surface/bulk regions.

In addition to changing the perovskite lattice constant and bandgap, our above analyses suggest that bromide affected the perovskite formation dynamics. Bromide and iodide have different solubility with the coordinating solvents used for perovskite deposition, such as dimethylformamide (DMF), dimethyl sulfoxide (DMSO), and gamma-Butyrolactone (GBL). We further used density functional theory (DFT) to calculate the interaction energies of PbXX′ (X and X′ denote either I or Br) species with DMSO. DMSO is partially removed by the antisolvent during spin coating, but the remaining residuals would form an adduct phase with PbXX′ and FAX to assist with the formation of the α-phase perovskite (*20*, *21*). From the optimized molecular configurations and bond distances shown in Fig. 3A and the interaction energies summarized in Fig. 3B, DMSO bonds most strongly with PbI_2_ (0.84 eV), followed by PbIBr (0.79 eV), and has the weakest bond with PbBr_2_ (0.77 eV), indicating that Br-rich species have lower solubility and therefore potentially nucleate first during supersaturation. Moreover, the formation energies for the PbXX′:DMSO adduct crystals were also calculated (fig. S16 includes the optimized crystal structures). The formation energies decreased as I was gradually replaced by Br (−0.18 eV for PbI_2_:DMSO, −1.10 eV for PbIBr:DMSO, and −1.32 eV for PbBr_2_:DMSO), suggesting that bromide-containing intermediate adduct phases are more thermodynamically favored to form. The surface energies between the triiodide perovskites with various A-site cations were also calculated to be much higher than that of the tribromide perovskites, regardless of the A-site cation (either PbX_2_ or X-site termination; fig. S17), indicating that Br species preferentially nucleate first, given that surface free energy increases the total free energy of nucleation. Taking the theoretical results altogether, bromide-containing species are therefore thermodynamically more favored to form compared to iodide-containing species and therefore complement the experimental observations that bromide-rich species formed first during the initial nucleation stage.

Both the experimental and theoretical results are complementary in showing that bromide drastically altered the perovskite formation dynamics by (i) promoting the initial nucleation of a bromide-rich species and (ii) introducing an anion exchange–like halide homogenization process, which is necessary to attain the final stoichiometry from the initial bromide-rich phase. The homogenization process is associated with a reconstruction of the PbX_6_ polyhedrons, with iodide diffusing toward the nuclei interior while bromide diffusing outward, or even halide migration across the interfaces. Unlike cation exchange or “molecular” exchange processes reported to assist the growth of high-quality thin-film perovskites (*22*, *23*), homogenization and self-diffusion of the anions during the nucleation and growth stages have rarely been studied for thin-film perovskites. Such processes have been shown to assist defect formation in other material systems, especially when interstitial diffusion and vacancy-assisted diffusion are the dominant pathways to homogenize the halides (*24*, *25*). We thus speculated that during the growth stage of FAMACsPb(I_0.8_Br_0.2_)_3_, these exchange and diffusion processes during homogenization promoted the formation of intrinsic defects associated with the halides, including vacancies, interstitials, and antisites. Our previous reports have shown the detrimental effects of point defects in perovskite materials, including V_I_, Pb_I_, and I_i_ (*9*, *26*, *27*). Complementarily, the strong PbI_2_ peak intensity observed by XRD for the fully annealed FAMACsPb(I_0.8_Br_0.2_)_3_ films suggests that the organic A-site cations were less stable and decompose more readily with higher Br% incorporation, which is supported by further theoretical calculations that show *V*_FA_ and FA_i_ were thermodynamically more favored to form when the Br% increases from 0 to 20% (fig. S18).

To investigate the defect density and nature of the films, we first compared the optoelectronic properties of the films using time-resolved PL (TRPL) measurements shown in Fig. 3C. A much shorter carrier lifetime of τ = 382.8 ns is observed for the FAMACsPb(I_0.8_Br_0.2_)_3_ film compared to the τ = 842.6 ns of FAMACsPbI_3_. In addition, we measured the quantified PL of the films and extracted the quasi-Fermi level splitting (qFLs) (Fig. 3D) based on a method we previously reported (*28*). The results show that the increasing qFLs did not track the bandgap increase with higher Br% incorporation. Twenty percent of Br incorporation resulted in the largest *V*_OC_ losses (*V*_OC,SQ_ − qFLs at 1 sun; Fig. 3E), and the voltage loss was reduced as Br% decreased. The large *V*_OC_ loss for the 0% Br sample is potentially due to α-phase degradation by exposure to high ambient humidity of ~40% relative humidity during sample measurement. Halide segregation during illumination was negligible for the FAMACsPb(I_0.8_Br_0.2_)_3_ film due to its highly optimized composition, as seen by its stable emission peak positions retained during continuous PL measurements (Fig. 3F).

**Fig. 4.**
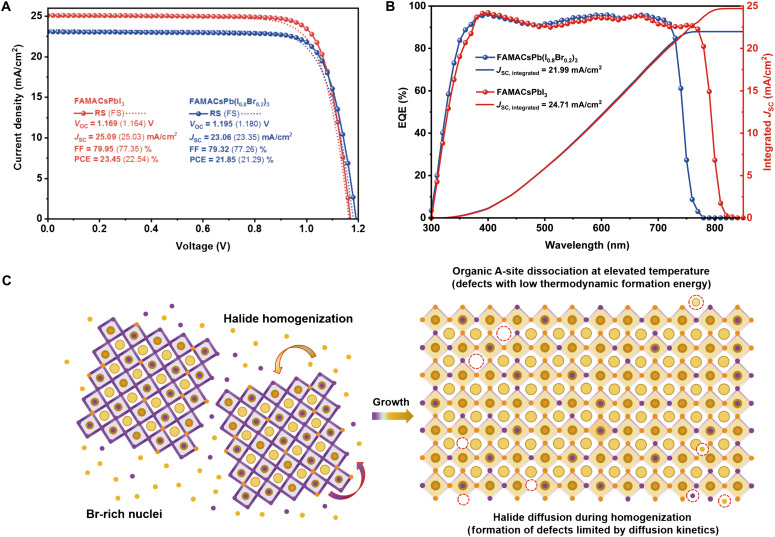
Solar cell devices and the proposed physical model. *J-V* characteristics (**A**) and EQE spectrum (**B**) of perovskite solar cell devices based on CsFAMAPb(I_0.8_Br_0.2_)_3_ and CsFAMAPbI_3_. (**C**) A hypothetic physical model of formation kinetics in mixed-halide perovskite with high Br% (left) and their potential role with defect physics (right). Violet dots stand for bromine, and yellow dots stand for iodine. Several but not all possible point defects were illustrated at the surface or in the bulk. For simplification purpose, only the halides were specified for Br as purple dots and I as yellow dots.

Positron annihilation spectroscopy (PAS) uses positively charged positrons that annihilate with negatively charged/neutral defects, capable of nondestructively probing the density and spatial distribution of defects in semiconductor thin films, and has been reported to be compatible with halide perovskite materials (*29*, *30*). Compared with admittance spectroscopy that also provides information about trap density, PAS could be carried out directly on bare perovskite films and is therefore independent of experimental variables that affect admittance spectroscopy results, such as the bandgap of the materials (which affects the device built-in potential) and the properties of the contacting transport materials (*31*). By controlling the kinetic energy of the incident positron beam and extracting the shape parameter from the gamma ray annihilation spectra (where a stronger gamma ray signal from positron annihilation could derive a larger shape parameter, indicating a higher density of negatively charged/neutral defects), a depth-resolved spectrum was obtained in Fig. 3G for the FAMACsPb(I_0.8_Br_0.2_)_3_ and FAMACsPbI_3_ films. We observed that the shape parameter for FAMACsPb(I_0.8_Br_0.2_)_3_ was clearly higher throughout the entire perovskite film, indicating a higher overall trap density. The surface region (shaded in green, 0 to 50 nm, Fig. 3G) contained a much higher density of surface defects, which will be detrimental to both the device performance and stability. FAMACsPb(I_0.8_Br_0.2_)_3_ also had a higher shape parameter in the perovskite bulk region (shaded in orange, 150 to 500 nm, Fig. 3G), indicating a higher trap density throughout the entire film. Crucially, this indicates that the defects formed through intrinsic processes (i.e., during perovskite formation and growth). In contrast, extrinsic processes (e.g., degradation) would have initiated from the film surface, preserving the bulk trap density of the film (*32*, *33*). Statistical analysis of the shape parameters comparing the surface and bulk regions is displayed in Fig. 3H. The eventual performance of the solar cell devices is a reflective consequence of the distinct defect physics and carrier dynamics of the films. As shown in Fig. 4A, devices based on both compositions exhibited >79% fill factor (FF), while the *J*_SC_ of FAMACsPb(I_0.8_Br_0.2_)_3_ (23.1 mA/cm^2^) was distinctly lower than that of FAMACsPbI_3_ (25.1 mA/cm^2^) due to the larger bandgap of the former. The external quantum efficiency (EQE) cutoffs (Fig. 4B) of the devices also matched well with the bandgaps estimated by the ultraviolet-visible absorption and PL measurements. The *V*_OC_ gain of FAMACsPb(I_0.8_Br_0.2_)_3_ over FAMACsPbI_3_, however, was insufficient to compensate for the bandgap increase, with *V*_OC_ values of 1.16 and 1.19 V (*V*_OC,SQ_ − *V*_OC_ of 0.13 and 0.20 V, *qE*_g_ − *V*_OC_ of 0.40 and 0.48 V), respectively. The solar cell performance results coincided with the film characterization results, where nonradiative recombination losses were more severe for the FAMACsPb(I_0.8_Br_0.2_)_3_ perovskite. A stability test was also carried out, from which the FAMACsPbI_3_ devices exhibited a much better long-term stability under illumination than FAMACsPb(I_0.8_Br_0.2_)_3_ devices after encapsulation (fig. S20). However, we found that the nonencapsulated devices of FAMACsPbI_3_ decayed at a much faster rate, which is consistent with our observation during the PL quantum yield (PLQY) tests regarding the fatal extrinsic instability (most likely due to moisture-induced phase transformation) of the FAMACsPbI_3_ perovskite. Detailed discussion on stability test is available in Supplementary Text.

## DISCUSSION

On the basis of all our experimental and theoretical observations and results, we summarize the physical model of the formation dynamics of WBG mixed-halide perovskites in Fig. 4C. The inclusion of bromide in the composition altered the perovskite crystallization pathway. A bromide-rich phase was more thermodynamically stable and thus nucleates first from solution during supersaturation. To attain the final target stoichiometry, during the growth stage, a halide homogenization process by diffusion gradually incorporates iodine into the expanding perovskite lattice. However, intrinsic defects formed readily during the homogenization process, and consequently, the final film had a higher defect density across the entire film depth, compared to the corresponding triiodide composition. Consequently, the WBG composition suffered from more severe nonradiative recombination losses and thus inferior optoelectronic properties and device performance. In this work, we correlated the complex perovskite formation dynamics with the defect physics and charge carrier dynamics of the resulting film. This study will guide the community to rethink the significance of precursor engineering and crystallization control for WBG mixed-halide perovskites toward more efficient and stable PVs based on these materials.

## MATERIALS AND METHODS

### Materials

DMF (99.8%), DMSO (99.7%), isopropanol (IPA; 99.7%), chlorobenzene (CB; 99.8%), acetonitrile (ACN; 99.8%), 4-tert-butyl pyridine (98%), lithium bis(trifluoromethanesulfonyl)imide (Li-TFSI; 99.95%), tris(2-(1*H*-pyrazol-1-yl)-4-*tert*-butylpyridine)cobalt(III) tri[bis(trifluoromethane)sulfonimide] [FK 209 Co(III) TFSI salt], and PbI_2_ (99.999%, perovskite grade) were purchased from Sigma-Aldrich. PbBr_2_ (99.999%), CsI (99.998%), deionized water, and SnO_2_ colloidal solution (15% in water) were purchased from Alfa Aesar. Formamidine iodide (FAI), formamidine bromide (FABr), methylammonium iodide (MAI), methylammonium bromide (MABr), and *n*-octylammonium iodide (OAI) were purchased from GreatCell Solar Materials (Australia). Methylammonium chloride (MACl) and Spiro-OMeTAD (99.8%) were purchased from Xi’an Polymer Light Technology (China). All the materials were used without further purification.

### Perovskite precursor preparation

#### FAMACs-based perovskites

CsI (35.8 mg/ml), FAI (192.6 mg/ml), MAI (22.3 mg/ml), and PbI_2_ (645.4 mg/ml) were dissolved in a DMF/DMSO = 80:20 solvent for the FAMACsPbI_3_ precursor. For FAMACsPb(I_0.8_Br_0.2_)_3_ precursor, CsI (35.8 mg/ml), FAI (168.6 mg/ml), PbI_2_ (516.3 mg/ml), MABr (31.4 mg/ml), and PbBr_2_ (102.8 mg/ml) were used. The final stoichiometries were (CsPbI_3_)_0.1_(FAPbI_3_)_0.8_(MAPbI_3_)_0.1_ and (CsPbI_3_)_0.10_(FAPbI_3_)_0.7_(MAPbBr_3_)_0.2_, respectively. Five to 50% Br precursors were prepared by partially or fully substitute MAPbI_3_ in the triiodide precursor by MAPbBr_3_ correspondingly.

#### FACs-based perovskites

CsI (71.7 mg/ml), FAI (192.6 mg/ml), and PbI_2_ (645.4 mg/ml) were dissolved in a DMF/DMSO = 80:20 solvent for the FACsPbI_3_ precursor. For FACsPb(I_0.83_Br_0.17_)_3_ precursor, CsI (71.7 mg/ml), FAI (192.6 mg/ml), PbI_2_ (480.8 mg/ml), and PbBr_2_ (115.6 mg/ml) were used. The final stoichiometries were FA_0.8_Cs_0.2_PbI_3_ and FA_0.8_Cs_0.2_Pb(I_0.83_Br_0.17_)_3_, respectively. Thirty mole percent of MACl were added in all perovskite precursors if without further specification. Ten to 50% Br precursors were prepared by maintaining the same cation ratio and only changing Br%.

### Perovskite film preparation and solar cell fabrication

All processes were done in a N_2_-filled glove box if without further specification. For the one-step FACs-perovskites and FAMACs-perovskites, the precursor solutions were casted to the substrate [either glass for in situ PL or glass/indium tin oxide (ITO)/SnO_2_ for solar cells] and spin-coated at 3500 rpm. CB was casted to the films at 45 s, and we led the spin continued for another 60 s. After spin coating, the samples would be immediately transferred to a 65°C hot plate for a 5-min preannealing and then transferred to a 150°C hot plate in ambient for 10 min of full annealing. No extra passivation treatment was carried out for the perovskite film characterizations.

For solar cell fabrication, SnO_2_ layer was prepared by spin coating a diluted SnO_2_ colloidal solution on precleaned glass/ITO substrates at 3000 rpm for 30 s followed with 30 min ambient annealing at 150°C. The perovskite layers were prepared on top of the SnO_2_ layer. For the best device performance, the passivation layer was done by spin-coating OAI (10 mM in IPA) at 5000 rpm followed with 1 min of annealing at 100°C. Twenty-five microliters of Spiro-OMeTAD solution prepared by mixing 85.8 mg of spiro-MeOTAD, 33.8 μl of 4-tert-butylpyridine, 19.3 μl of Li-TFSI (520 mg ml^−1^ in ACN) solution, and 17.9 μl of FK 209 Co(III) TFSI salt (375 mg ml^−1^ in ACN) in 1 ml of CB was spin-coated at 3000 rpm on the perovskite surface. After aging in dry air overnight, 120-nm gold was then evaporated to form the metal electrodes.

### Thin films and device characterizations

For in situ PL measurement, a homebuilt platform consists of a 532-nm laser diode and an Ocean Optics Spectrometer (Flame) coupled with optical fibers, optical filters, and several planoconvex lenses described in our previous reports were used (*34*, *35*). The power density of the laser was approximately 100 mW/cm^2^ as the intensity reaching the sample could vary as adjusting the setup. Integration time of each spectra was 100 ms during the measurement. Calibrated measurements for qFLs were carried out in another homebuilt PL setup also with 532-nm excitation. The laser beam is quantified by a charge-coupled device camera and a power meter to calculate the equivalent number of suns from the photon flux. The wavelength dependence of the spectrometer and the collection optics is corrected using a commercial calibration lamp with known spectrum. For the TRPL measurement, a PicoHarp 300 with time-correlated single-photon counting capabilities excited by the 640-nm pulse laser diode (PLD 800B, PicoQuant) with a repetition frequency of 200 kHz. A biexponential decay fitting model was used to extract the charge carrier lifetime. For the XRD measurement, an x-ray diffractometer (PANalytical) with Cu Kα radiation at a scan rate of 3° min^−1^ was used, and the contact angle was obtained by VCA optima 100. All XRD measurements were carried out in ambient environment after transferring the samples out from the N_2_-filled glove box. Scanning electron microscopy was captured by a Nova Nano 230 system. PAS measurement was carried out at the Washington State University positron beam. Experimental details and spectra analysis methods have been fully discussed in our previous publication (*27*). An empirical formula of $d=\frac{40\;E_{k}^{1.6}}{\rho }\;(\frac{\text{nm}∙g}{{\text{cm}}^{3}{\text{keV}}^{1.6}})$ was used to convert the kinetic energy of incident positron (*E*_k_) into depth profile. The fraction of the annihilated gamma ray signal in a narrow central region that increase in relative intensity at positron trap sites was determined as the shape parameter. The *J-V* characteristics were measured with a Keithley 2401 meter under the illumination of an Oriel Sol3A class AAA solar simulator (Newport) that simulated AM 1.5 G spectrum (100 mW/cm^2^) calibrated with an NREL-certified Si photodiode with KG-5 filter. The EQE was measured by an Enlitech designed system under AC mode without bias light.

### DFT calculations

All quantum chemical calculations of interaction energies between DMSO and PbXX′ were performed using Gaussian 16.1 (*36*). The initial structures used in computations were constructed using GaussView 6.0.16. PbXX′ was positioned at various locations around the DMSO, and the geometry of each resulting DMSO:PbXX′ complex was optimized with the ωB97X-D density functional and the 6-31+G(d,p) basis set for C, H, O, S, and Br atoms and LANL2DZ basis set for Pb and I atoms. Optimized geometries were verified as minima (zero imaginary frequencies) by frequency calculations at the same level of theory. Interaction energies were computed using the same level of theory and using the following equation: ∆*E*_int_ = *E*_(DMSO:PbXX^′^)_ − [*E*_DMSO_ + *E*_PbXX^′^_]. Molecular structures were visualized and rendered using Mercury 4.3.1 (Build 273956). (*37*–*39*)

All first-principle calculations for bulk and slab systems are carried out using the Vienna Ab initio Simulation Package (VASP) code.(*40*, *41*). A revised Perdew-Burke-Ernzerhof generalized gradient approximation (PBEsol) (*42*, *43*) was used for the exchange correlation including a dispersion correction using Grimme’s DFT-D3 scheme (*44*, *45*). PBEsol functional has been introduced to improve the equilibrium properties of solids and surfaces (*46*). Valence-core interactions were described by projector augmented wave pseudopotentials (*47*). Plane wave expansions with kinetic energies up to 300 eV were chosen as the basis set for all geometry optimization and energy calculations. Both atomic positions and cell dimensions were optimized using a conjugate gradient algorithm until all Hellman-Feynman forces are smaller than 0.02 eV/Å. Γ-center *k*-point mesh (4 × 4 × 4 and 4 × 4 × 1) was adopted for Brillouin zone sampling in bulk and surface calculations, respectively.

For formation energies of DMSO:PbXX′ adduct crystals, we used the following expression: Δ*H*(DMSO : PbXX^′^) = *H*(DMSO : PbXX^′^) − [*H*(DMSO) + *H*(PbXX^′^)], where the first, second, and third terms in the right hand side are the energies of DMSO:PbXX′, DMSO, and PbXX′, respectively. We considered XX′ = I_2_, IBr, and Br_2_ and used the monoclinic DMSO in all cases, and the crystal structures of DMSO:PbXX′ and PbXX′ are based on orthorhombic DMSO:PbI_2_ and hexagonal PbI_2_ crystals, respectively.

For surface energy calculations, the 2 × 2 × *L* surfaces were formed along (001) by periodic slabs including 9 to 11 atomic layers for a surface separated by 10 to 15 Å of vacuum. To characterize the formation and stability of the ABX_3_ surfaces, we use surface energies described as follows: we first calculate the cleavage energy of the clean surface

$$\sigma^{\text{clv}}=\frac{1}{2}[E_{\text{slab}}^{\text{unrel}}-NE_{\text{bulk}}+\sum_{i}n_{i}\mu_{i}]$$ (1)

where the first term is the energy of the unrelaxed slab, μ*_i_* is the chemical potentials, *n_i_* is the number of *i*th unit removed, and *E*_bulk_ = μ*_A_* + μ_Pb_ + 3μ*_X_* is the energy per formula unit of the bulk perovskite. The relaxation energy is then calculated from

$$\sigma^{\text{rl}}=E_{\text{slab}}^{\text{rel}}-E_{\text{slab}}^{\text{unrel}}$$ (2)

where the first and second terms are the total energy of the relaxed and unrelaxed slabs, respectively. The surface energy is calculated by γ = (σ^clv^ + σ^rel^)/*A*, where *A* is the area of the surface. For the APbX_3_ perovskite, *A* = FA, MA, or Cs and *X* = Br or I were calculated independently. For this study, we considered PbX_2_-terminated surfaces.

Formation energies of the defects in FAPb(I*_x_*Br_1−*x*_)_3_ in a charge-state *q* is calculated from

$$\Delta H_{d}(q)=E_{d}(q)-E_{\text{pr}}+\Sigma_{i}n_{i}\mu_{i}\prime +q(E_{F}+E_{\text{VBM}})$$ (3)

where *E*_d_(*q*) is the total energy of the system with defect and *E*_pr_ is the pristine system. μ*_i_*′ = μ_0*i*_ + μ*_i_* is the chemical potential, which includes intrinsic μ_0*i*_ and growth condition–dependent term μ*_i_*. *n_i_* is the number of *i*th unit (i.e., atom or molecule) exchanged with the reservoir to form the defect. *E*_F_ is the Fermi energy, and *E*_VBM_ is the energy at the valence band maximum. Following Freysoldt’s correction scheme, the energy corrections arising from the spurious interactions due to the finite-size are included in Eq. 3.

To determine the chemical potentials required for formation energy of a FAPb(I*_x_*Br_1−*x*_)_3_

$$\mu_{\text{FA}}+\mu_{Pb}+3[{x\mu }_{I}+(1-x)\mu_{\text{Br}}]=\Delta H_{1}$$ (4)

should be satisfied under thermodynamic equilibrium growth conditions, where Δ*H*_1_ is the formation energy of FAPb(I*_x_*Br_1−*x*_)_3_. In this study, we considered *x* = 1, 0.8, and 0. To avoid the formation of undesired phases

$$\mu_{\text{FA}}+\mu_{I}<\Delta H(\text{FA}I),\mu_{\text{FA}}+\mu_{Br}<\Delta H(\text{FABr})$$ (5)

$$\mu_{\text{Pb}}+2\mu_{I}<\Delta H(\text{Pb}I_{2}),\mu_{\text{Pb}}+2\mu_{\text{Br}}<\Delta H(\text{Pb}{\text{Br}}_{2})$$ (6)

conditions should also be satisfied where appropriate. The formation energies in the right hand sides of Eqs. 5 and 6 are calculated from $\Delta H(\text{FABr})=H(\text{FABr})-[H(\text{FA})+\frac{1}{2}H(Br_{2})]$, $\Delta H(\text{FAI})=H(\text{FAI})-[H(\text{FA})+\frac{1}{2}H(I_{2})]$, Δ*H*(PbI_2_) = *H*(PbI_2_) − [*H*(Pb) + *H*(I_2_)], and Δ*H*(PbBr_2_) = *H*(PbBr) − [*H*(Pb) + *H*(Br_2_)]. Here, to calculate each energy term, we used the respective structures: FABr and FAI in the rock-salt phases, PbI_2_ and PbBr_2_ in the hexagonal phases, FA in the body-centered cubic phase, Pb in the cubic phase, and I_2_ and Br_2_ in the gas phase. We considered the Pb-rich growth conditions in all chemical potential calculations.
