# Supplementary material for: Performance-limiting formation dynamics in mixed-halide perovskites
Source: Sci Adv. 2021 Nov 10;7(46):eabj1799. doi: 10.1126/sciadv.abj1799 (PMC8580316; doi:10.1126/sciadv.abj1799)
Supplement: Supplementary file 1 — Supplementary Text Figs. S1 to S20 [file sciadv.abj1799_sm.pdf]

Science Advances
AAAS

Supplementary Materials for

Performance-limiting formation dynamics in mixed-halide perovskites

Tianyi Huang, Shaun Tan, Selbi Nuryyeva, Ilhan Yavuz*, Finn Babbe, Yepin Zhao, Maged Abdelsamie, Marc H. Weber, Rui Wang, Kendall N. Houk, Carolin M. Sutter-Fella*, Yang Yang*

*Corresponding author. Email: yangy@ucla.edu (Y.Y.); csutterfella@lbl.gov (C.M.S.-F.); ilhan.yavuz@marmara.edu.tr (I.Y.)

Published 10 November 2021, *Sci. Adv.* **7**, eabj1799 (2021)
DOI: 10.1126/sciadv.abj1799

This PDF file includes:

Supplementary Text
Figs. S1 to S20

Supplementary Text

1. Discussion on XRD results for FAMACs and FACs-based perovskites

High-resolution XRD measurements were carried out for both the FAMACs and FACs-based perovskites. The as-cast and fully annealed films with both tri-iodide and mixed-halide compositions were measured and shown in **Fig. S1** and **S2**. The diffraction intensity was also extracted for the (001) perovskite peak and the PbI_2 peak. In both the FAMACs and FACs systems, the mixed-halide films exhibited larger 2θ peak positions compared with the tri-iodide ones, which correspond to smaller lattice constants. Meanwhile, the film crystallinity of the mixed-halide films was apparently weaker, potentially due to the homogenization process, because the pure tri-bromide films exhibited superior crystallinity by itself (see discussions below). The PbI_2 characteristic peak (or more accurately, the PbX_2 peak in the mixed-halide case) was only detected in the fully annealed films and was much stronger in the mixed-halide case. From the SEM images of the fully annealed films in **Fig. S4**, we also observed large amounts of plate-like crystalline PbI_2 at the surface of $FAMACsPb(I_{0.8}Br_{0.2})_3$. Considering that all films were prepared by the exact same deposition process, we speculate that the organic A-site cations were less stable and decomposed to form the residual lead halide binaries during thermal annealing for the mixed-halide films.

2. Discussion on XRD results for $MAPb(I_XBr_{1-X})_3$, $FAPb(I_XBr_{1-X})_3$, $FAPb(I_XBr_{1-X})_3$ with MACl, $FAMACsPb(I_XBr_{1-X})_3$, and $FACsPb(I_XBr_{1-X})_3$.

High-resolution XRD measurements were carried out for $MAPb(I_XBr_{1-X})_3$ perovskites (where X= 1, 0, 0.9, 0.8 or 0.5 for $MAPbI_3$, $MAPbBr_3$, $MAPb(I_{0.9}Br_{0.1})_3$, $MAPb(I_{0.8}Br_{0.2})_3$, and $MAPb(I_{0.5}Br_{0.5})_3$, respectively) in order to completely exclude any effects by the cations and MACl, even though MA-based perovskites are not the optimum composition to achieve high performance WBG mixed-perovskite PVs. The MA-based films are deposited following the same solvent combination (DMF/DMSO = 80/20) and antisolvent deposition methods as the FAMACs perovskites. It should also be noted that the cation could tremendously affect the growth pathway also, for example, $MAPbI_3$ will form a robust MAI-DMSO-PbI_2 intermediate phase before annealing, but the perovskite phase remains dominant in the as-cast films of the FAMACs and FACs systems (especially for tri-iodide perovskites). The diffraction peaks at the 2θ range of 13.5° to 15.5° was extracted and shown in Fig. S8 for the as-cast films, pre-annealed films (65°C for 1 min), and fully annealed films (100°C for 10 min).

The (110) peak for $MAPbI_3$ and the (100) peak for $MAPbBr_3$ are easily identified in **Fig. S5A** and **S5B** at 14.2° and 14.9°, respectively. The peak intensity is extremely low for the as-cast $MAPbI_3$ film because of the dominant MAI-DMSO-PbI_2 intermediate phase at this stage. The diffraction peaks for $MAPbI_3$ shifted negligibly during their perovskite growth when comparing the as-cast film, pre-annealed film, and the fully annealed film. In contrast, $MAPbBr_3$ showed very strong crystallinity even without any heat treatment, consistent with our DFT results that their formation is thermodynamically favored. In all mixed-halide cases, we observed dramatic shifting of their 2θ peak positions towards lower values, indicating that there is a clear Br% change during perovskite growth.

Similar to $MAPb(I_XBr_{1-X})_3$, we have also characterized $FAPb(I_XBr_{1-X})_3$ (in the case with or without MACl additive, **Fig. S6** and **S7**) to show these observations were not limited to MA

systems. However, we found except for FAPbBr3, MACl is essential for $FAPbX_3$-based compositions to form their a-phase at lower temperature as no diffraction signals from the (100) perovskite peak of $FAPbX_3$ could be observed before annealing at 150 ˚C. Interestingly, adding MACl directly assisted the formation of the α-phase perovskite, but a segregated growth feature was still observed and more severe than the MA system such as $FAPb(I_{0.8}Br_{0.2})_3$, in which homogeneous halide merely formed after full annealing.

MACl is another factor that need to be excluded to confirm the existence of homogenization process. We further characterized $FAMACsPb(I_XBr_{1-X})_3$ and $FACsPb(I_XBr_{1-X})_3$ (without MACl, additive, **Fig. S8** and **S9**) films, where only two halides were involved. Similarly, the shifting of perovskite (100) peak from a higher 2θ to a lower 2θ was again observed, which suggested the existence of the halide homogenization process without MACl. Segregated phases were observed for fully annealed $FAMACsPb(I_{0.5}Br_{0.5})_3$ and $FAMACsPb(I_{0.5}Br_{0.5})_3$ films here, indicating MACl could be helpful assist the formation of homogeneous films during annealing.

3. Discussion on in-situ PL results for FACs-based perovskites

The in-situ PL measurements were carried out for the FACs-based perovskites. The complete contour plots during both the spin-coating and annealing periods are shown in **Fig. S1**3, and the extracted parameters (peak position, intensity, FWHM) in **Fig. S14**.

Both the peak position shifts and the time elapsed before the peak intensity and FWHM stabilize showed consistent trends with the $FAMACsPbX_3$ perovskites. For $FA_{0.8}Cs_{0.2}Pb(I_{0.83}Br_{0.17})_3$, the peak shift was observed to be ΔE_1= 0.246 eV during spin-coating and ΔE_2= 0.095 eV during annealing. For $FA_{0.8}Cs_{0.2}PbI_3$, these values were as low as ΔE_1= 0.176 eV and ΔE_2= 0.054 eV. Similar to the results for the $FAMACsPbX_3$ perovskites, the energy shift is larger with Br inclusion. The excess growth stage is still observed and can be quantified by a biexponential decay model (t_1= 11.81s, t_2=35.70s for $FA_{0.8}Cs_{0.2}Pb(I_{0.83}Br_{0.17})_3$, and t_1=t_2=20.20 s for $FA_{0.8}Cs_{0.2}PbI_3$). That it is less apparent than the $FAMACsPbX_3$ perovskites might be attributed the incorporation of MA to further assist the process originated from Br% addition.

4. Discussion on the stability test of the perovskite devices

Considering the extrinsic instability (mainly from moisture induced α-to-δ phase transformation, especially for $FAMACsPbI_3$) and the intrinsic instability (mainly from the defects and ion migration, for both compositions), a set of stability test of non-encapsulated and encapsulated devices were carried out (shown in **Fig. S20**). We found without encapsulation, $FAMACsPbI_3$ went through a rapid decay after the first 24h, and quickly lost all PV performance as the photo-active α-phase easily degraded to δ phase. In comparison, the $FAMACsPb(I_{0.8}Br_{0.2})_3$ still retained ~30% of its original PCE after 300h aging even without encapsulation, indicating relatively good phase stability due to the lowered tolerance factor from the excess $MAPbBr_3$ incorporated.

Encapsulating the device prevent the penetration of oxygen and moisture that induces the degradation of perovskite devices via many pathways. In contrasting with the devices without encapsulation, $FAMACsPbI_3$ devices showed a much better stability improvement. After ~300h, it retained approximately 77% of their original performance, while encapsulated $FAMACsPb(I_{0.8}Br_{0.2})_3$ devices went through a much faster decay rate where ~50% of their initial

performance were lost at the end of the stability test. These results again indicated the better intrinsic stability of tri-iodide perovskite than the mixed-halide WBG perovskite potentially due to their distinct formation dynamics and defect physics.

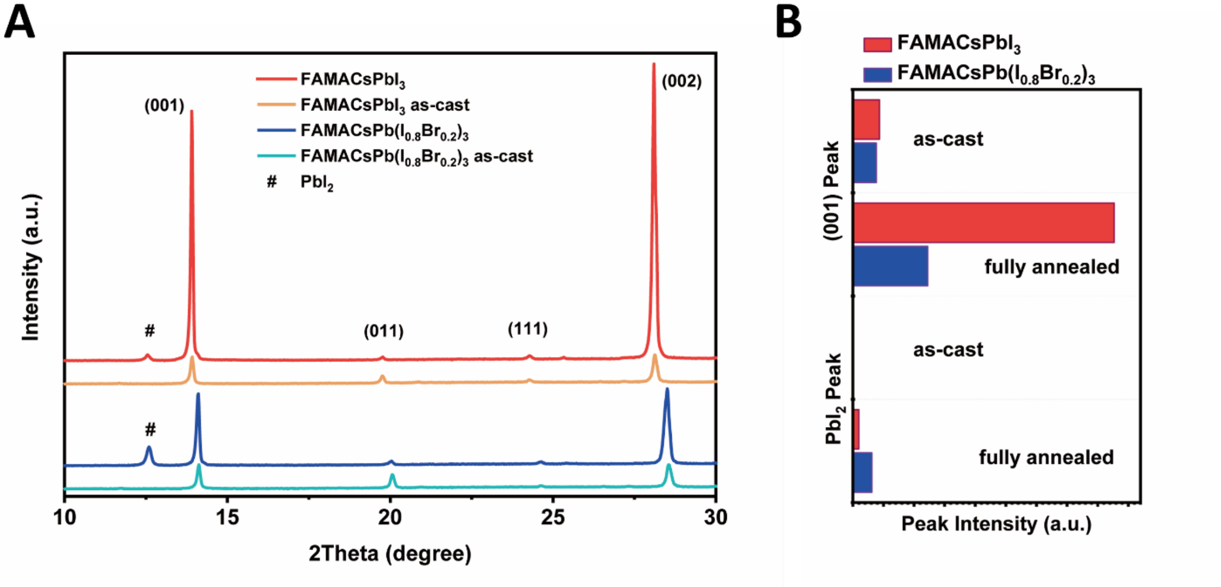

Fig S1. XRD results of the as-cast and fully annealed perovskite films of FAMACsPbX$_3$. (A) The XRD patterns of the as-cast and fully annealed perovskite films of FAMACsPbX$_3$. (B) The extracted peak intensities of the (001) peak of perovskite and the PbI_2 peak.

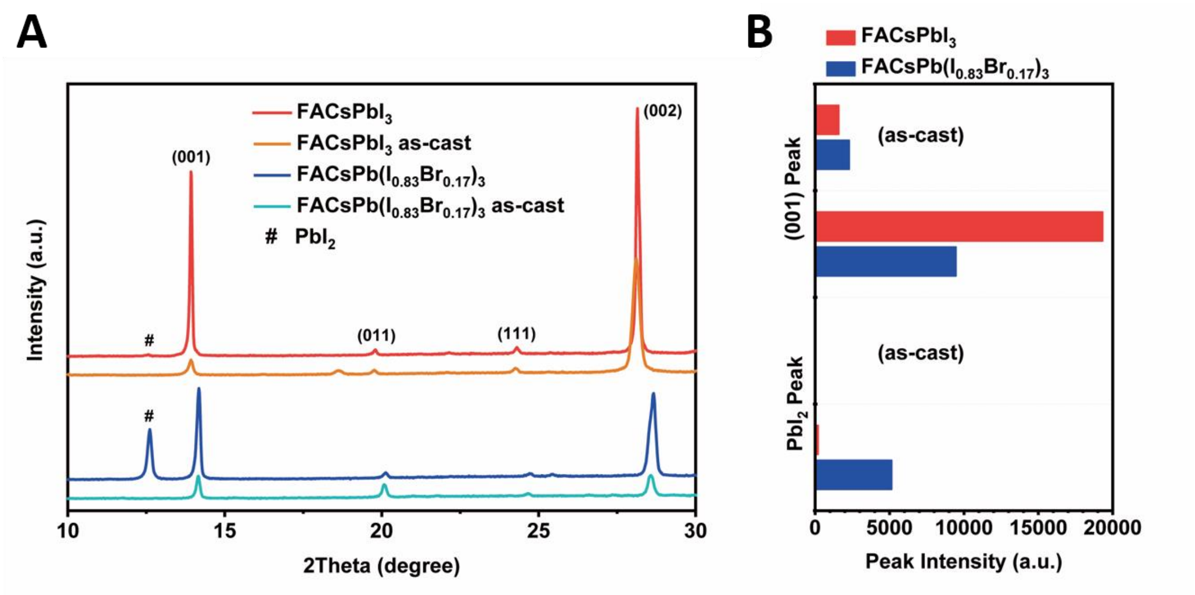

Fig S2. XRD results of the as-cast and fully annealed perovskite films of $FACsPbX_3$. (A) The XRD patterns of the as-cast and fully annealed perovskite films of $FACsPbX_3$. (B) The extracted peak intensities of the (001) peak of perovskite and the PbI_2 peak.

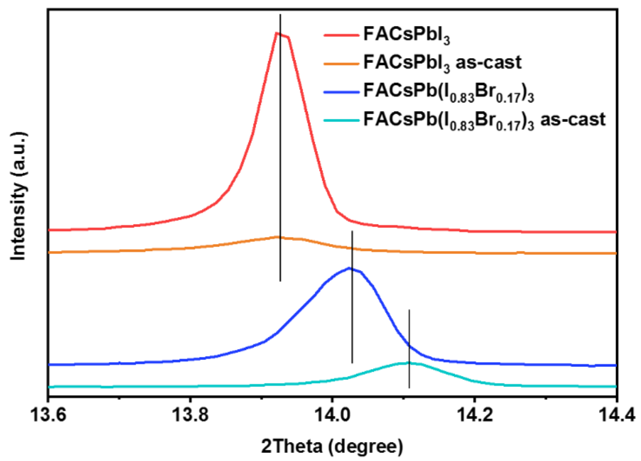

Fig S3. XRD spectra of (001) peak of $FACsPbX_3$ films.

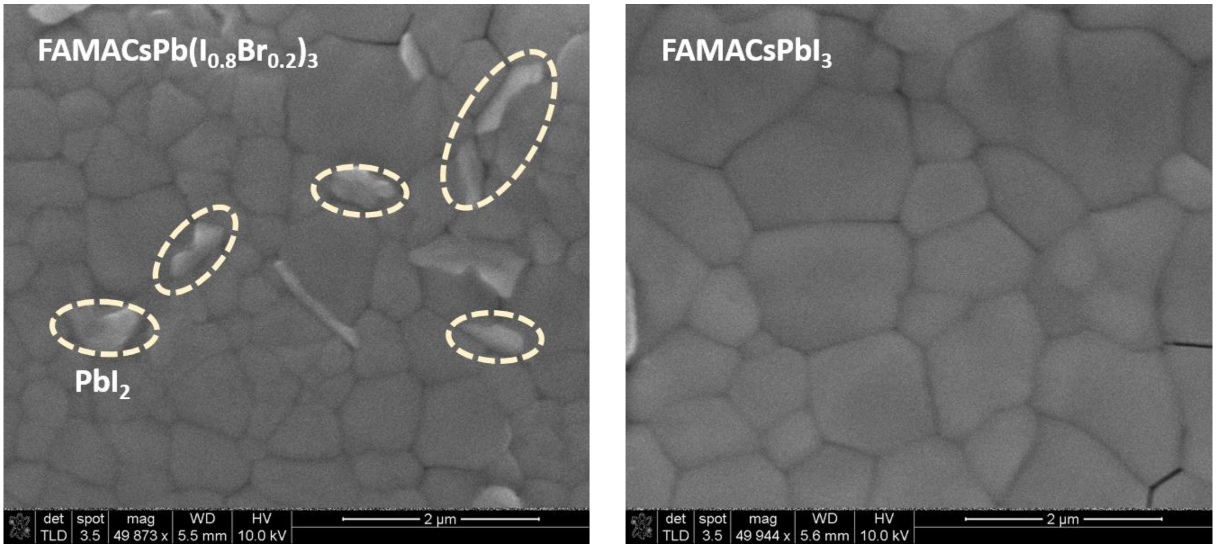

Fig S4. SEM image of $CsFAMAPb(I_{0.8}Br_{0.2})_3$ and $CsFAMAPbI_3$. Flakes-shape PbI_2 can be observed on the $CsFAMAPb(I_{0.8}Br_{0.2})_3$ surface. For $CsFAMAPbI_3$ prepared as exact same annealing condition, surface PbI_2 can be hardly observed.

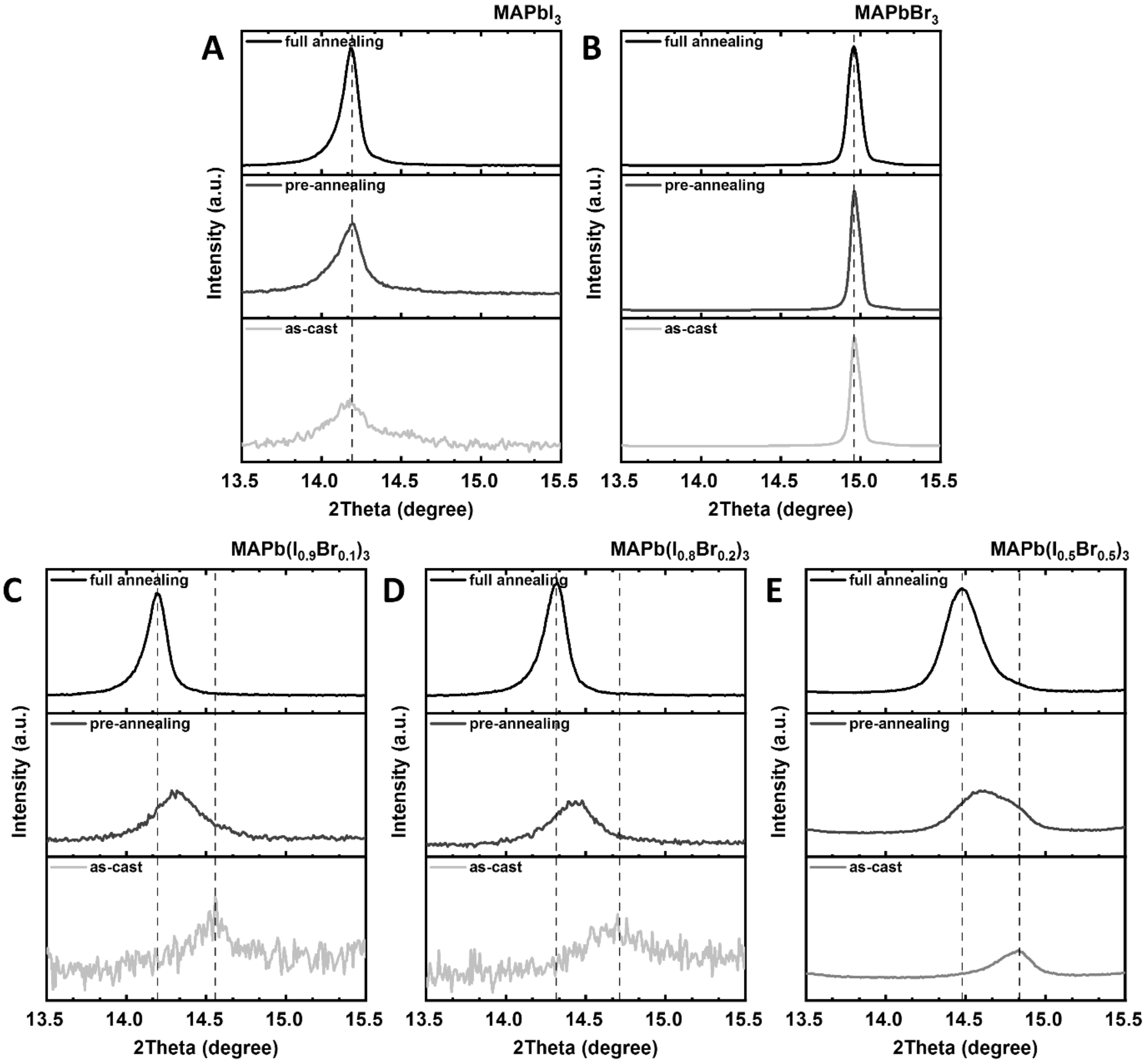

Fig S5. XRD results of MAPb(I_XBr_{1-X})$_3$ films. High resolution diffraction patters at the 2θ range of 13.5° to 15.5° of the as-cast, pre-annealed (65°C for 1 min), and fully annealed (100°C for 10 min) MAPb(I_XBr_{1-X})$_3$ films (**without MACl additive**). From A-E are X=1, 0, 0.9, 0.8 and 0.5, respectively.

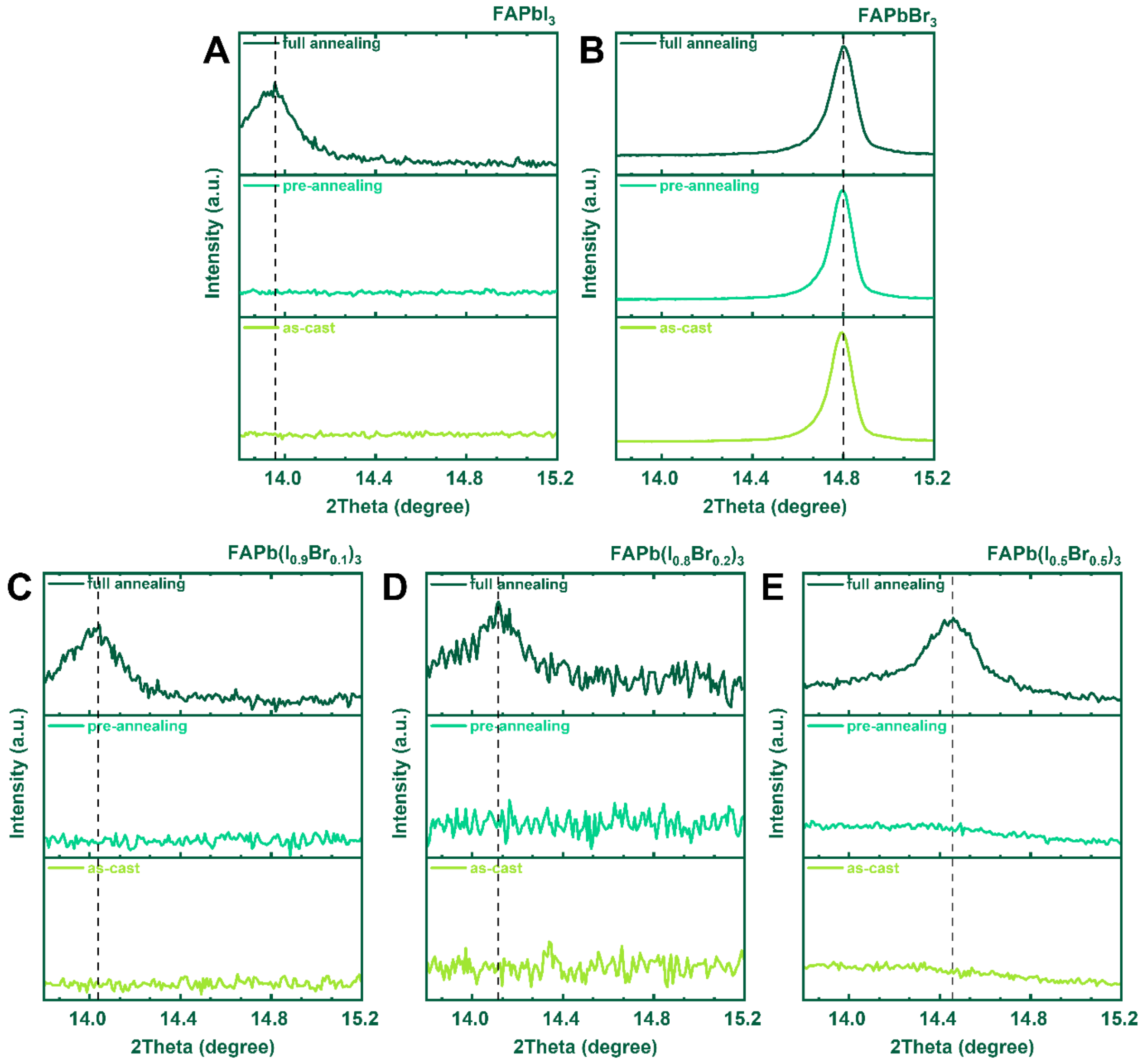

Fig S6. XRD results of FAPb(I_XBr_{1-X})$_3$ films. High resolution diffraction patters at the 2θ range of 13.5° to 15.2° of the as-cast, pre-annealed (65°C for 1 min), and fully annealed (150°C for 10 min) FAPb(I_XBr_{1-X})$_3$ films (**without MACl additive**). From A-E are X=1, 0, 0.9, 0.8 and 0.5, respectively.

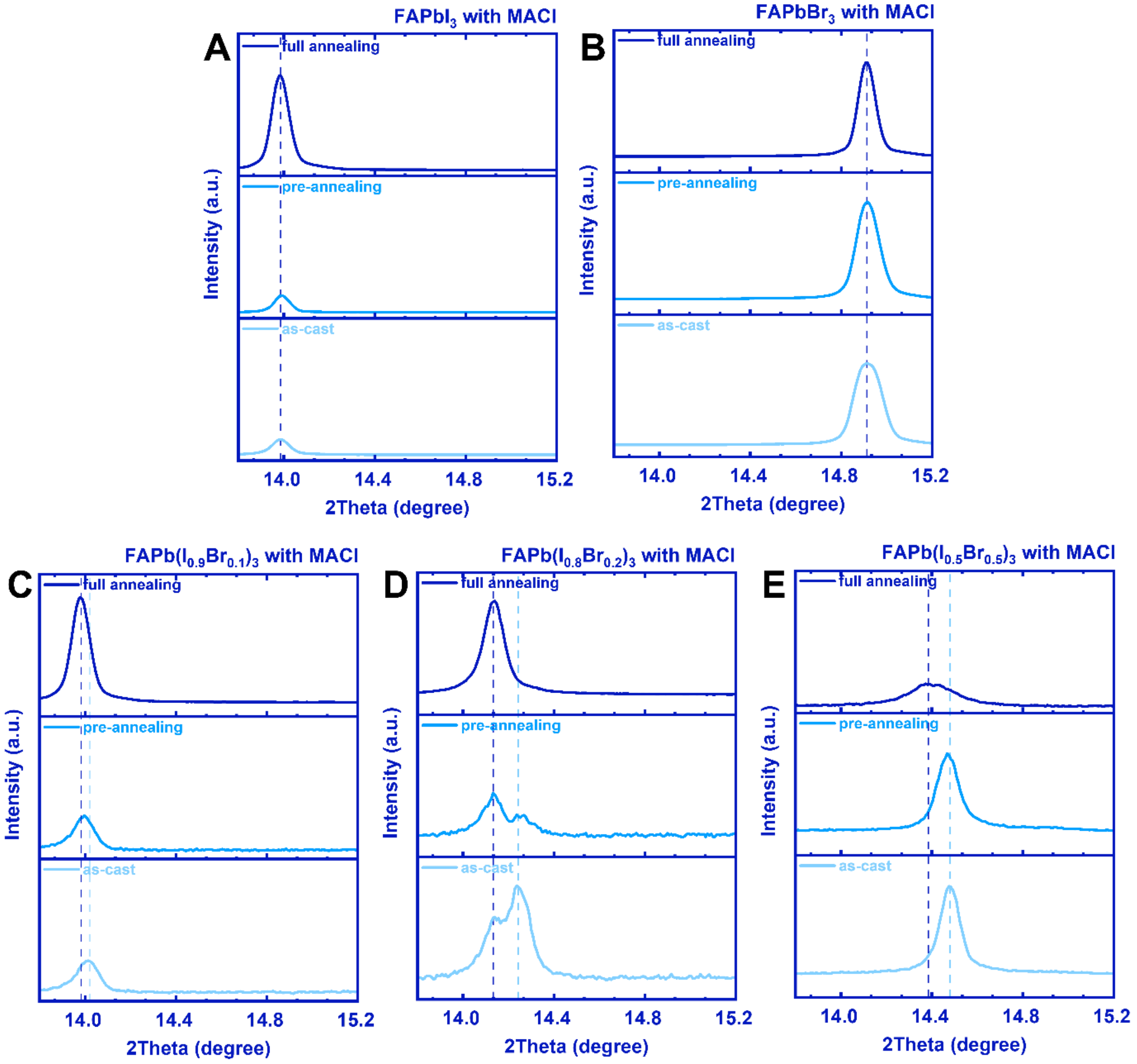

Fig S7. XRD results of $FAPb(I_XBr_{1-X})_3$ films with MACl additive. High resolution diffraction patters at the 2θ range of 13.5° to 15.2° of the as-cast, pre-annealed (65°C for 1 min), and fully annealed (150°C for 10 min) $FAPb(I_XBr_{1-X})_3$ films (**with MACl additive**). From A-E are X=1, 0, 0.9, 0.8 and 0.5, respectively.

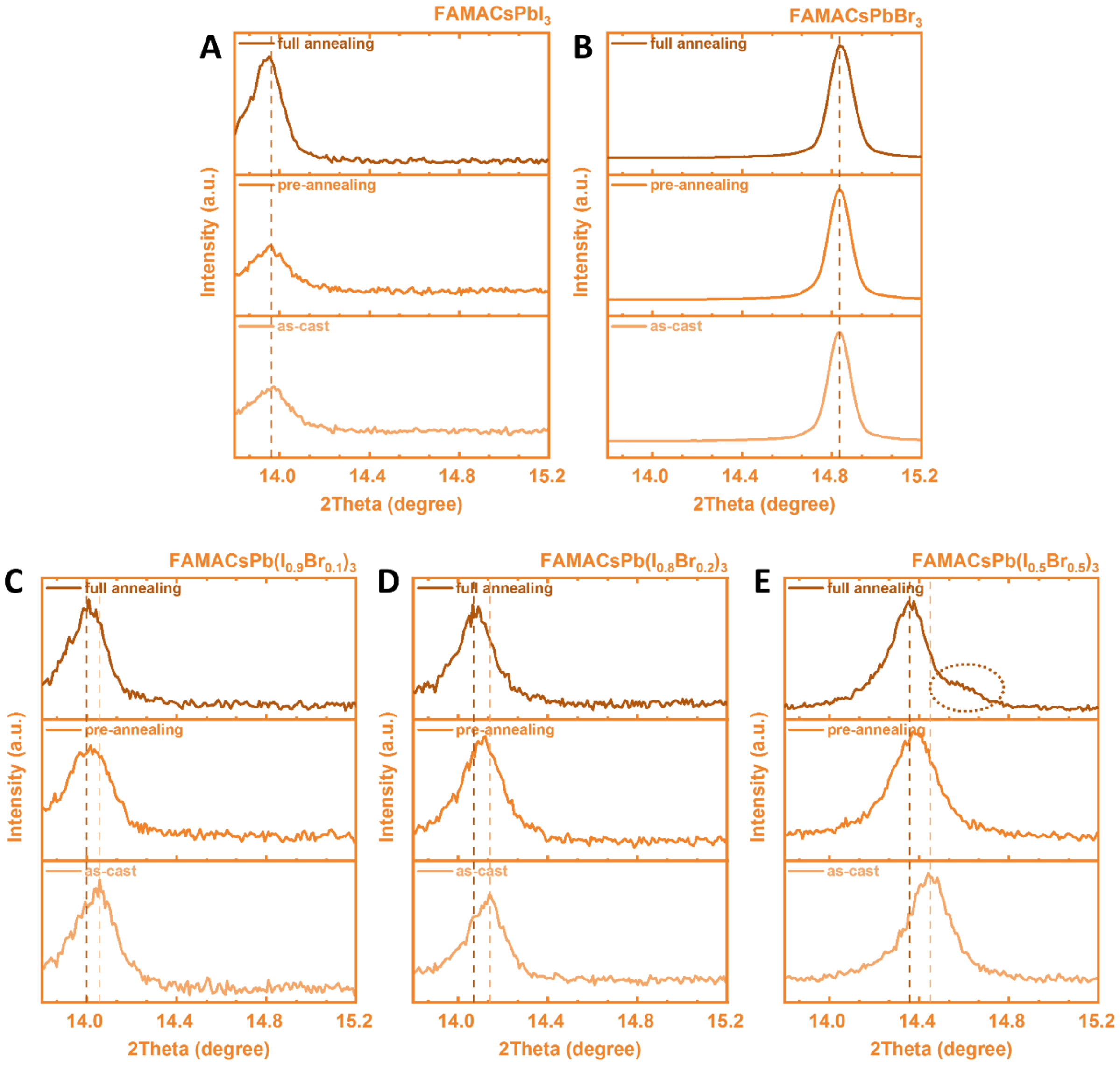

Fig S8. XRD results of FAMACsPb(I_XBr_{1-X})$_3$ films. High resolution diffraction patters at the 2θ range of 13.5° to 15.2° of the as-cast, pre-annealed (65°C for 1 min), and fully annealed (150°C for 10 min) FAMACsPb(I_XBr_{1-X})$_3$ films (**without MACl additive**). From A-E are X=1, 0, 0.9, 0.8 and 0.5, respectively.

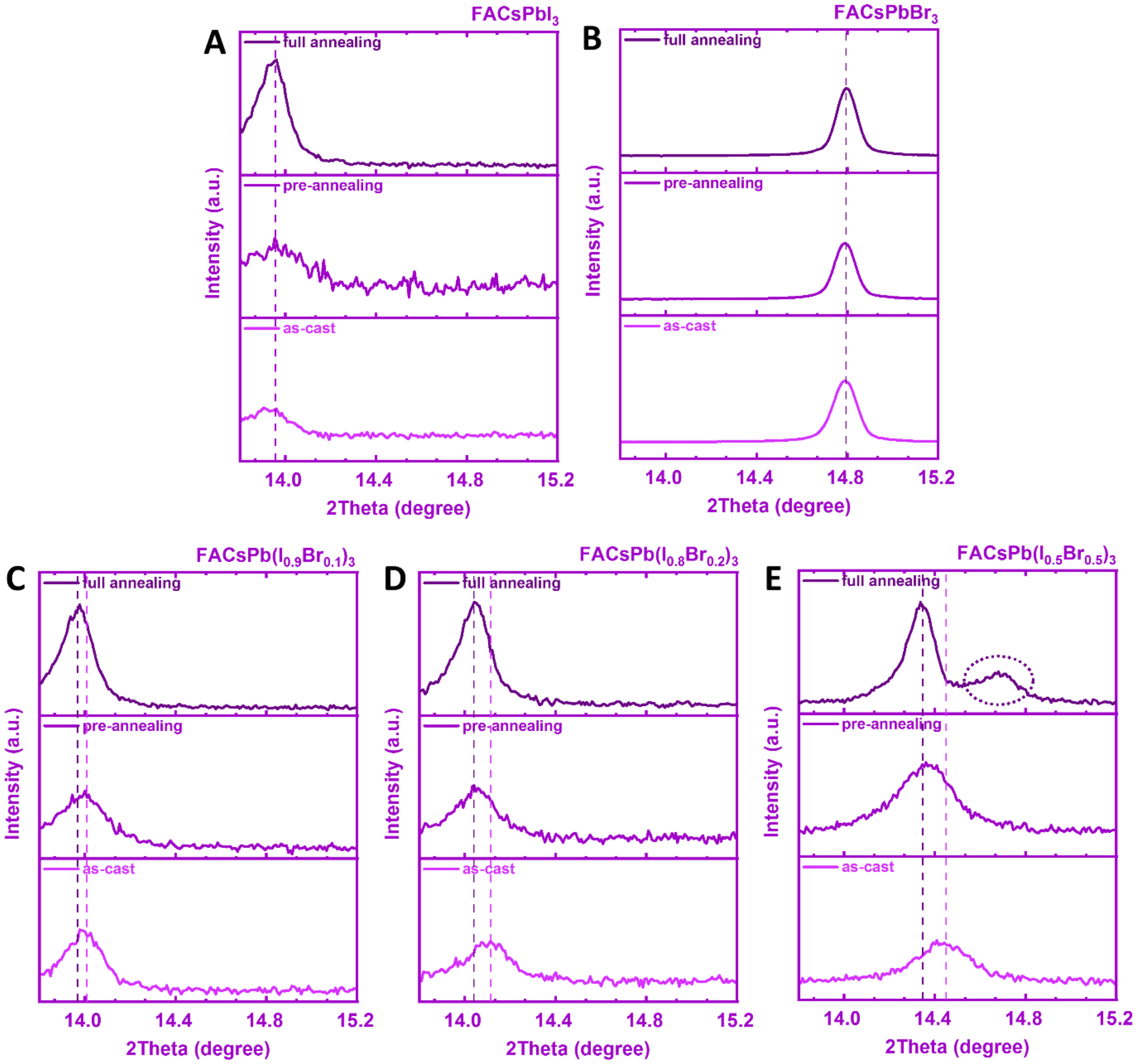

Fig S9. XRD results of $FACsPb(I_XBr_{1-X})_3$ films. High resolution diffraction patters at the 2θ range of 13.5° to 15.2° of the as-cast, pre-annealed (65°C for 1 min), and fully annealed (150°C for 10 min) $FACsPb(I_XBr_{1-X})_3$ films (**without MACl additive**). From A-E are X=1, 0, 0.9, 0.8 and 0.5, respectively.

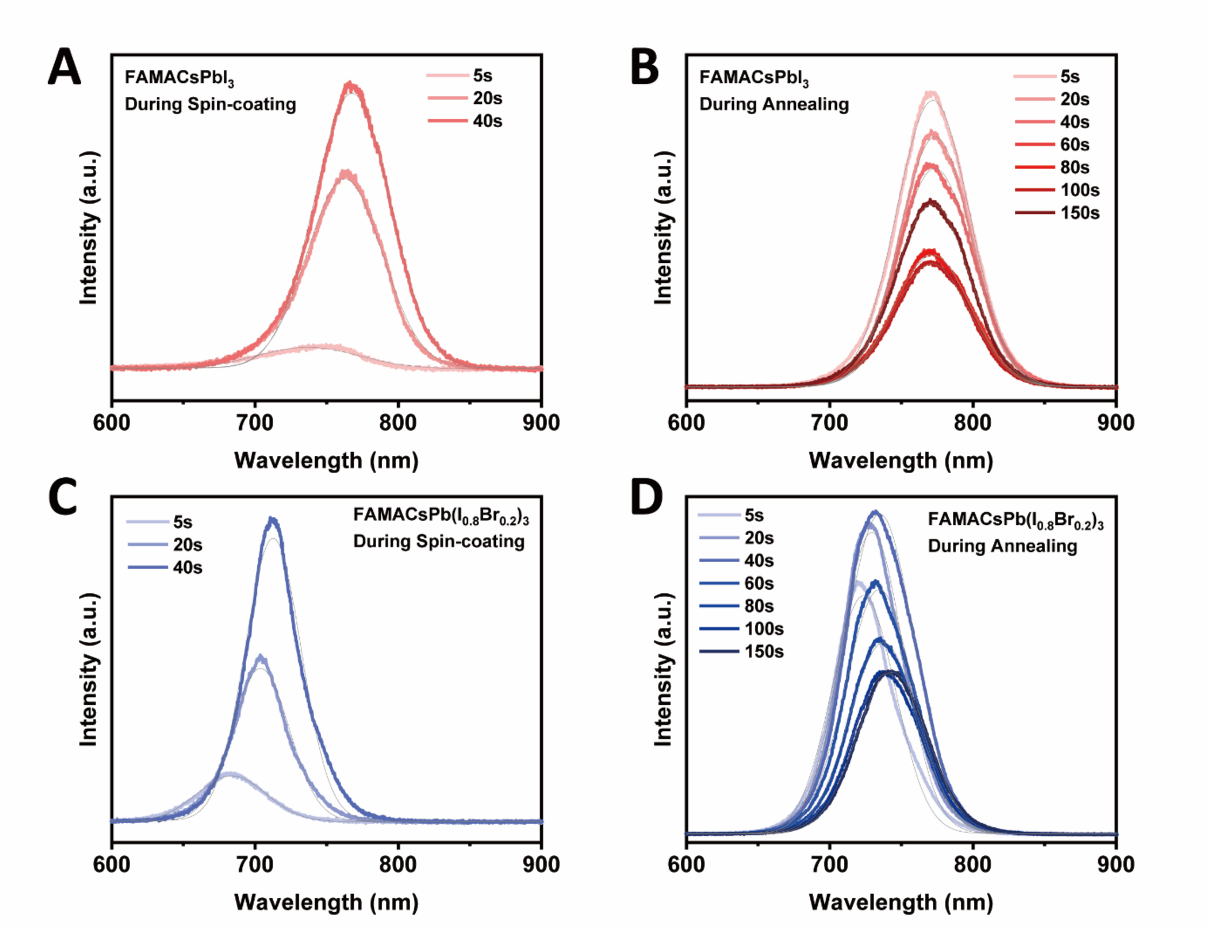

Fig. S10. Selected spectra from in-situ PL measurements. During spin-coating (A and C) and annealing (B and D) for $FAMACsPbI_3$ and $FAMACsPb(I_{0.8}Br_{0.2})_3$. Gaussian fitting results shown in grey lines.

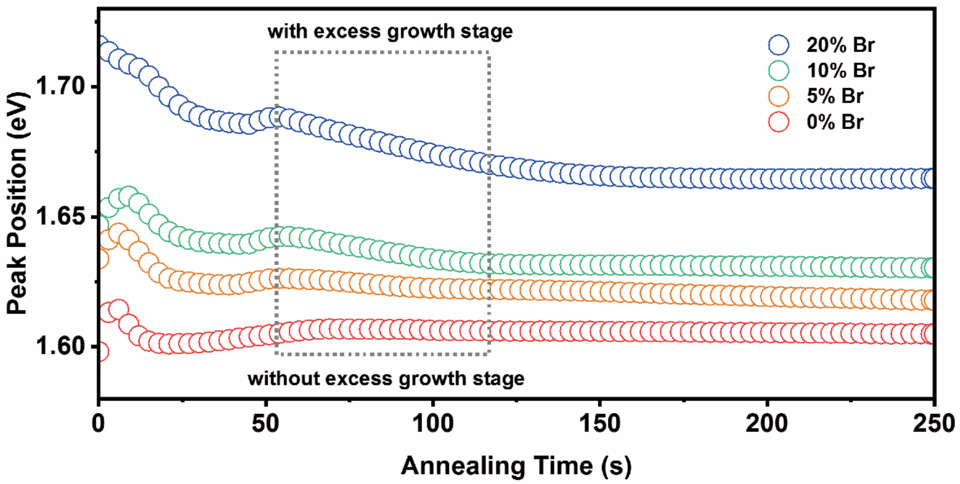

Fig. S11. The extracted values of emission peak position for FAMACsPb(I,Br)$_3$ perovskite with Br% of 20%, 10%, 5% and 0%. A slight rise in peak position observed before ~50 s is result from the enhance and broadening of the PL signal (details disccued in **Fig. S13**).

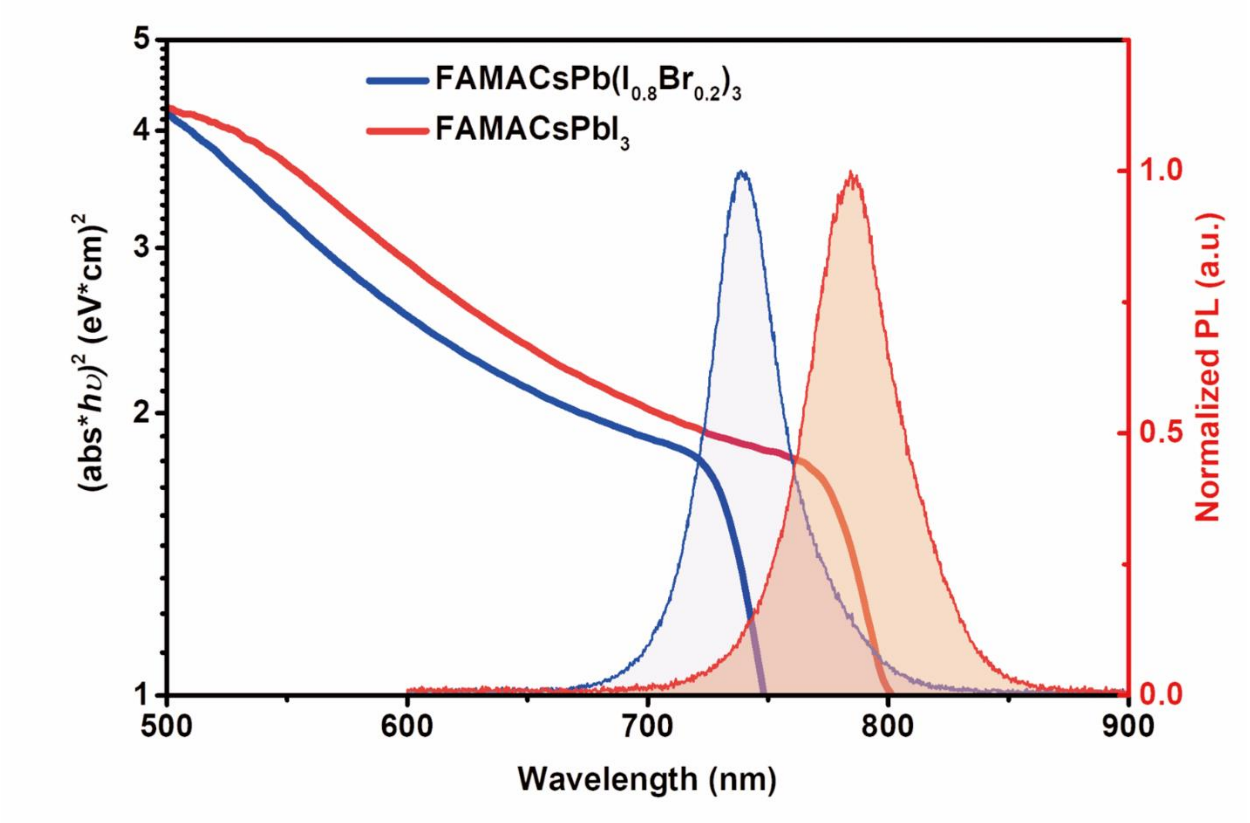

Fig. S12. UV-Absorption curve (in Tauc plot) and static PL spectra of the fully annealed perovskite films.

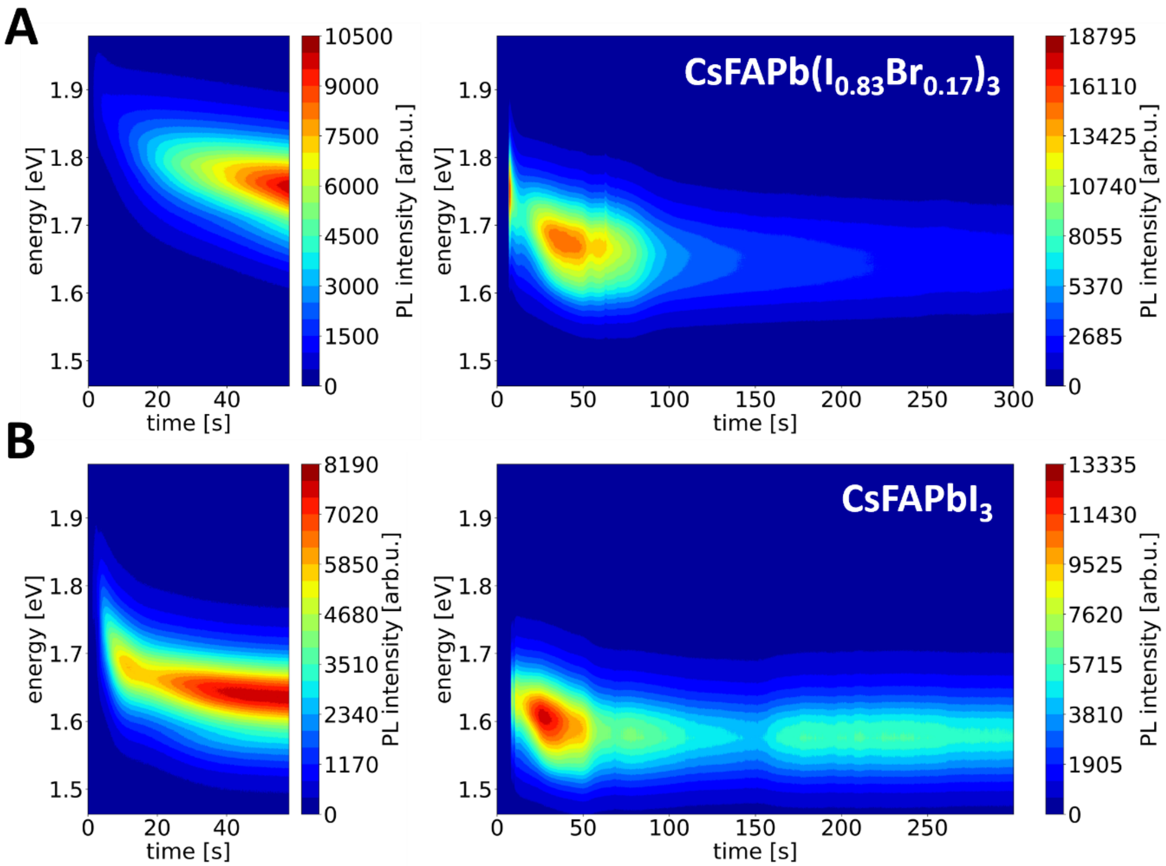

Fig. S13. The contour plot of the captured PL spectra during the growth of $FACsPbX_3$ perovskites. (A) $FACsPb(I_{0.83}Br_{0.17})_3$ and (B) $FACsPbI_3$ films during spin-coating (left) and annealing (right).

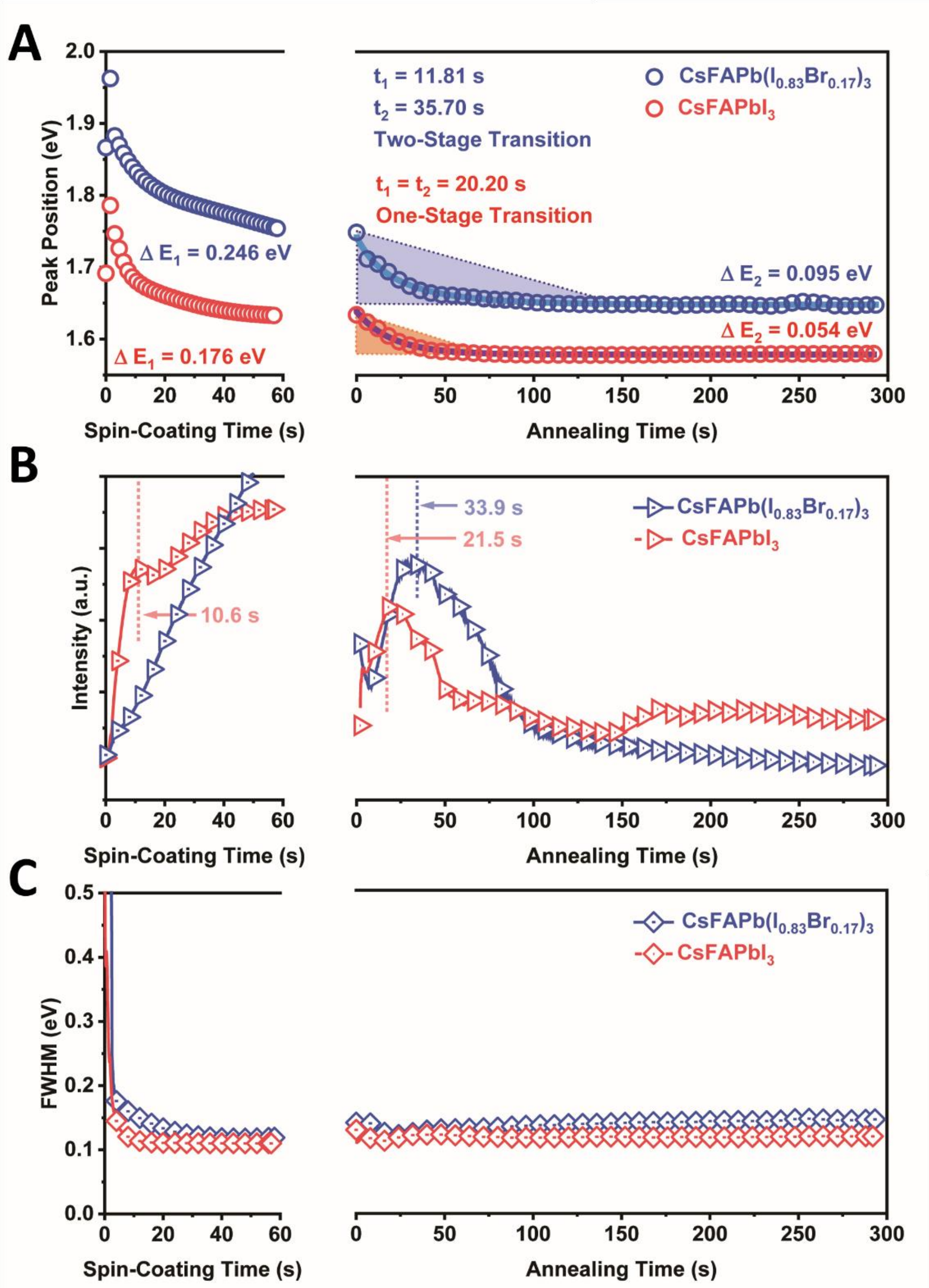

Fig. S14. The extracted values of emission peak position (A), PL intensity (B), and FWHM (C) from the in-situ PL measurements for $FACsPbX_3$ perovskites. By fitting the peak position signals during annealing with a biexponential decay model, it was found that the $FACsPb(I_{0.83}Br_{0.17})_3$ film also exhibited an excess growth stage (two-stage transition) while the $FACsPbI_3$ film still had a one-stage transition.

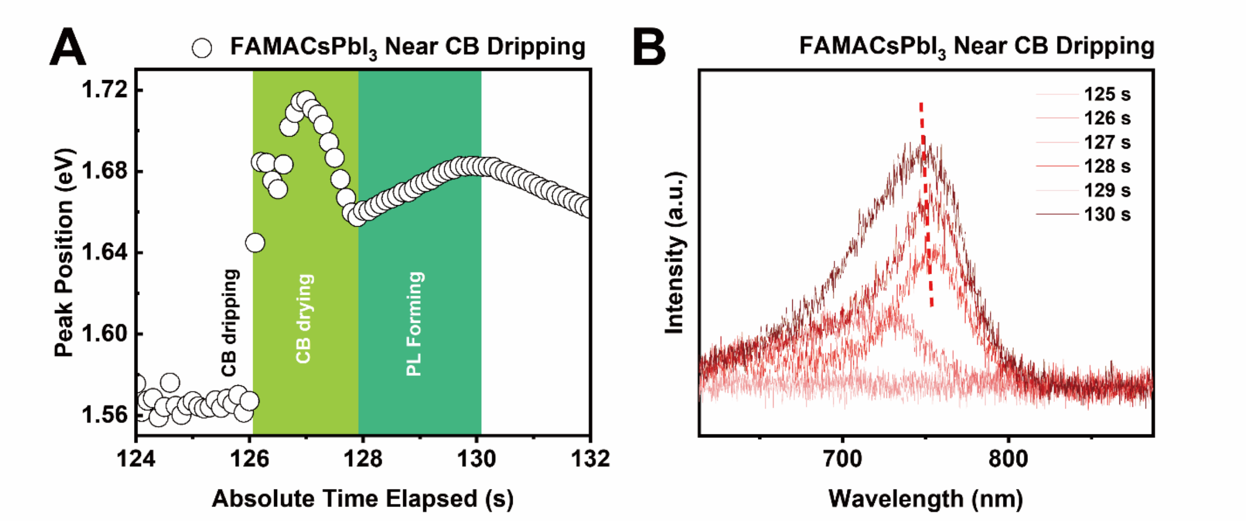

Fig. S15. PL signals at *absolute* time scale near CB casting. The extracted values of emission peak position (A) at the absolute time elapsed and the corresponding raw PL spectra (B) near the moment of CB (anti-solvent) casting replotted from the original dataset of Fig. 2D (left slab). The PL transition near CB dripping could be rationally divided into three stages: (I). Before CB dripping that no PL could be observed (refer to the spectrum of 125s). At this stage, the “Peak Position” values were simply extracted from gaussian fitting of the background spectra. (II). CB with boiling point as high as 132˚C will take seconds to fully spin-off and evaporate from the film surface after dripping. At this “CB drying” stage, quasi-stable nanoscale surface perovskite nuclei with strong quantum confinement effect could form but could also re-dissolve into the residual solvent in the wet precursor film bulk (even though the overall thermodynamics would move to the direction of nuclei growth). The composition of these nuclei can far deviate from the precursor and with much higher Br%. As a result, the actual PL signal started to form from extremely lower intensity and at much higher energy level (also refer to Fig. 2E), while the fitted PL peak position value also rose from that of the background spectra. (III). After the CB and residual solvent being mostly removed, and PL signal starts to form as perovskite grows initiated from the surface. An obvious rising and broadening of PL was observed at this stage, and with the rapid increase in PL intensity and FWHM, peak position naturally went through slight increased (blue shift) as an increase in charge carrier density populating in the excited states. These electronic dynamics upon nucleation occurs much faster than the bandgap change due to halide migration and homogenization, thus dominated the peak position changes at this stage.

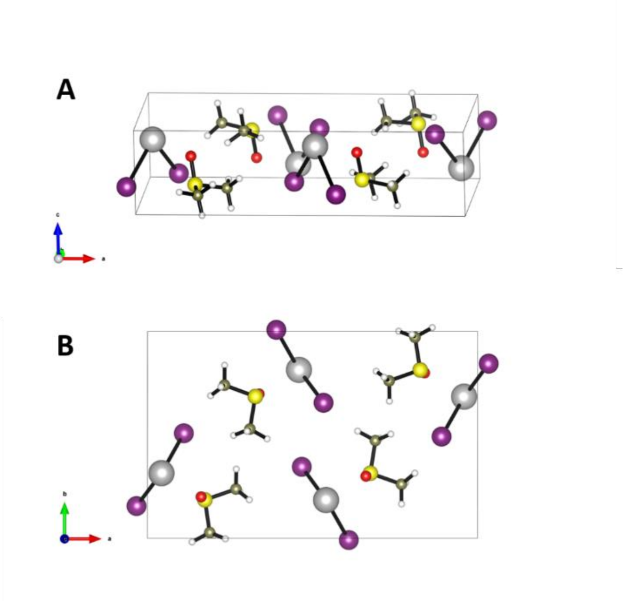

Fig S16. Side (A) and top (B) view of the optimum crystal structure of DMSO:PbXX' adduct. X sites were assumed to be I in the structure.

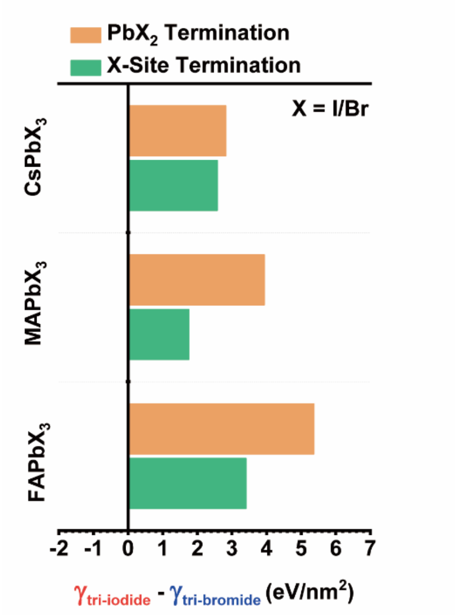

Fig S17. The calculated results of surface energy difference in tri-iodide perovskites and tri-bromide perovskites with cations to be Cs, MA, or FA.

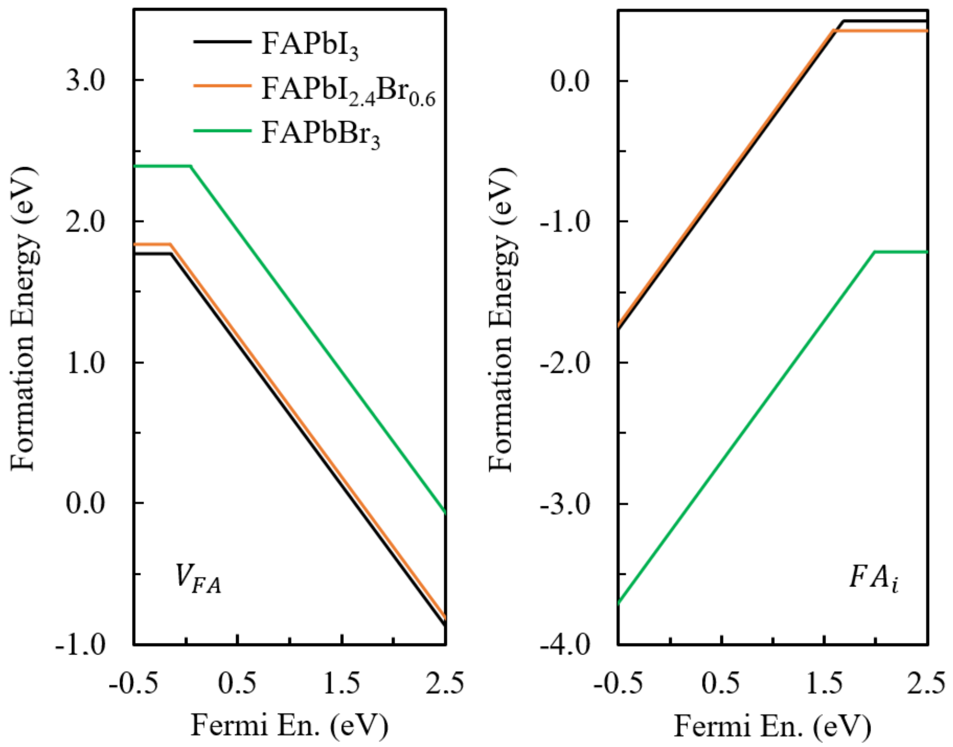

Fig S18. Fermi energy (E_F) dependence of the defect formation energy of FA vacancy and FA interstitial point defects. $FAPbI_3$ (orange line), $FAPb(I_{0.8}Br_{0.2})_3$ (black line) and $FAPbBr_3$ (green line). E_F = 0 was set at VBM.

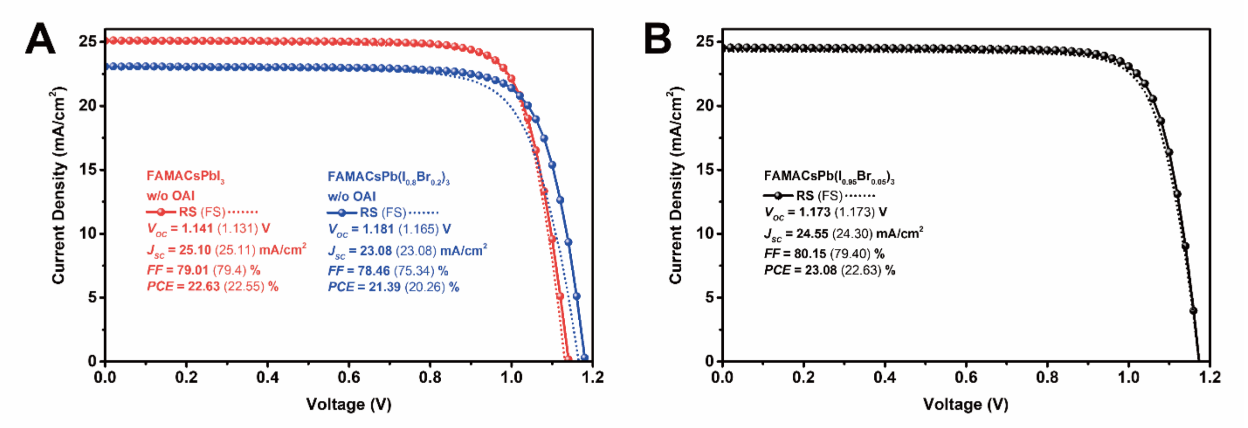

Fig S19. Supplementary *J-V* characteristics of perovskite solar cell devices. (A) $CsFAMAPb(I_{0.8}Br_{0.2})_3$ and $CsFAMAPbI_3$ without OAI surface treatment; (B) $CsFAMAPb(I_{0.95}Br_{0.05})_3$.

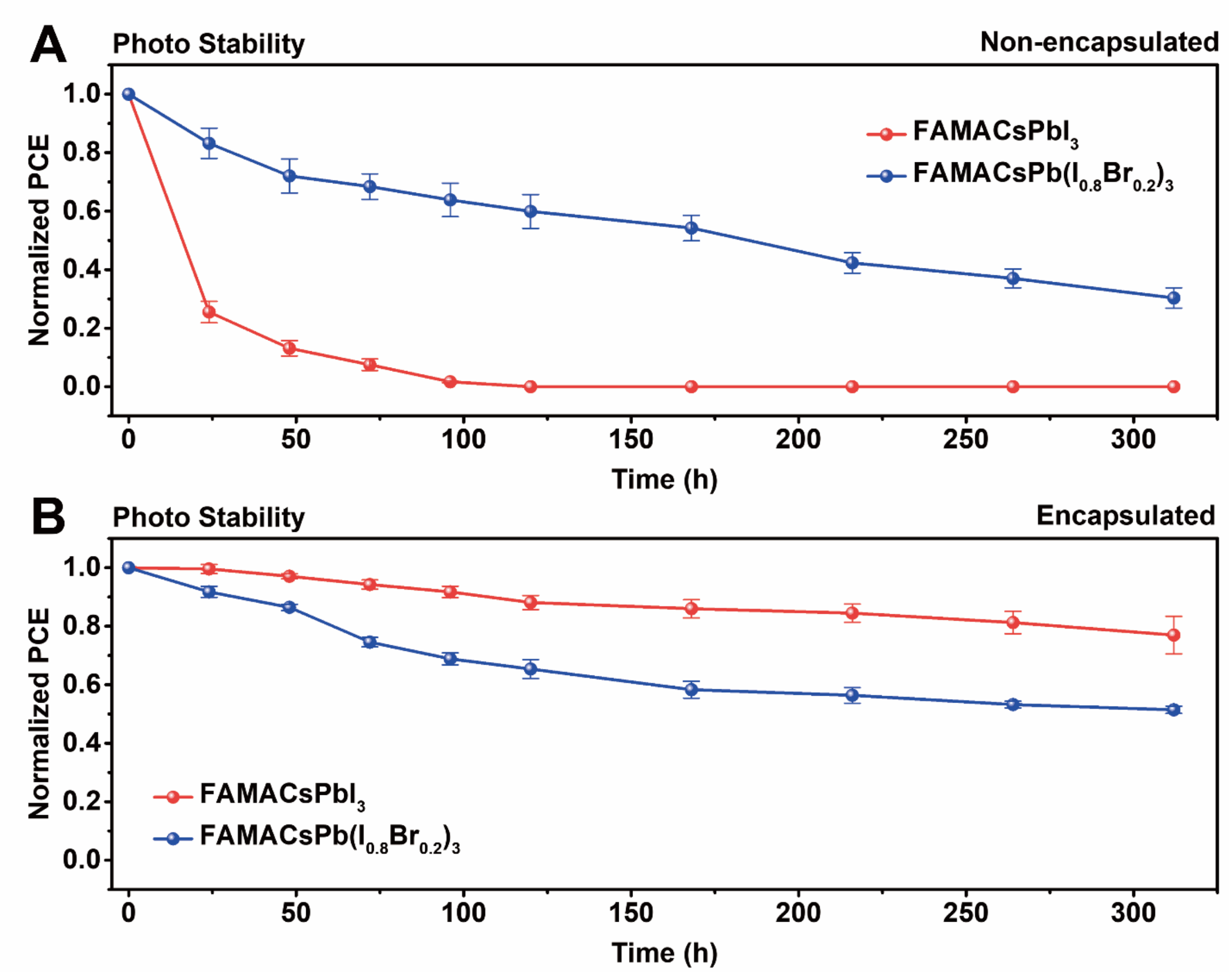

Fig S20. Photo stability test of perovskite solar cells. The devices are based on $FAMACsPbI_3$ and $FAMACsPb(I_{0.8}Br_{0.2})_3$ under (A) non-encapsulated or (B) encapsulated conditions. Data was obtained from the average of 6 devices of each condition. The samples were aged under illumination of 90 (±10) mW/cm^2 at open-circuit and cooled by a mini fan during the test.
